# HREM, RNAseq and Cell Cycle Analyses Reveal the Role of the G2/M-Regulatory Protein, WEE1, on the Survivability of Chicken Embryos during Diapause

**DOI:** 10.3390/biomedicines10040779

**Published:** 2022-03-27

**Authors:** Narayan Pokhrel, Olga Genin, Dalit Sela-Donenfeld, Yuval Cinnamon

**Affiliations:** 1 Agriculture Research Organization, The Volcani Center, Department of Poultry and Aquaculture Science, Bet Dagan 50250, Israel; narayan.pokhrel@mail.huji.ac.il (N.P.); olga.genin5654@gmail.com (O.G.); 2 The Robert H. Smith Faculty of Agriculture, Food and Environment, Koret School of Veterinary Medicine, The Hebrew University of Jerusalem, Rehovot 7610001, Israel

**Keywords:** chicken embryonic diapause, cell cycle, G2/M transition, chicken embryonic blastoderm, WEE1, high-resolution episcopic microscopy (HREM), RNAseq, mitosis

## Abstract

Avian blastoderm can enter into diapause when kept at low temperatures and successfully resume development (SRD) when re-incubated in body temperature. These abilities, which are largely affected by the temperature and duration of the diapause, are poorly understood at the cellular and molecular level. To determine how temperature affects embryonic morphology during diapause, high-resolution episcopic microscopy (HREM) analysis was utilized. While blastoderms diapausing at 12 °C for 28 days presented typical cytoarchitecture, similar to non-diapaused embryos, at 18 °C, much thicker blastoderms with higher cell number were observed. RNAseq was conducted to discover the genes underlying these phenotypes, revealing differentially expressed cell cycle regulatory genes. Among them, *WEE1*, a negative regulator of G2/M transition, was highly expressed at 12 °C compared to 18 °C. This finding suggested that cells at 12 °C are arrested at the G2/M phase, as supported by bromodeoxyuridine incorporation (BrdU) assay and phospho-histone H3 (pH 3) immunostaining. Inhibition of WEE1 during diapause at 12 °C resulted in cell cycle progression beyond the G2/M and augmented tissue volume, resembling the morphology of 18 °C-diapaused embryos. These findings suggest that diapause at low temperatures leads to *WEE1* upregulation, which arrests the cell cycle at the G2/M phase, promoting the perseverance of embryonic cytoarchitecture and future SRD. In contrast, *WEE1* is not upregulated during diapause at higher temperature, leading to continuous proliferation and maladaptive morphology associated with poor survivability. Combining HREM-based analysis with RNAseq and molecular manipulations, we present a novel mechanism that regulates the ability of diapaused avian embryos to maintain their cytoarchitecture via cell cycle arrest, which enables their SRD.

## 1. Introduction

The avian embryo at oviposition, termed blastoderm, corresponds to the blastocyst stage in mammalian embryos [1]. However, unlike mammalian embryos, growth and development of the avian embryo can be arrested for a prolonged duration at low temperature prior to gastrulation in a phenomenon known as diapause [2]. In nature, the ability to diapause allows for obtaining synchronous hatching of eggs laid in a clutch. The poultry industry utilizes the diapause phenomenon to store eggs prior to incubation [3,4]. The ability to diapause for long duration, and the capacity to successfully resume development (SRD) thereafter, highly depends on the temperature at which the embryos are kept during diapause. We have recently demonstrated that embryos stored at 12 °C have a greater chance to SRD following prolonged diapause than embryos stored at 18 °C [3]. The reasons for this variability could be explained by the activation or inhibition of different molecular and cellular processes at the corresponding diapause temperatures.

At oviposition, the blastoderm consists of 60,000–130,000 cells distributed in an outer ring termed the area opaca (AO), which forms extra embryonic structures, and an inner disk known as the area pellucida (AP), which is composed of an epithelium layer, termed epiblast, that gives rise to the embryo proper [3,5,6,7,8]. From the epiblast, single cells normally detach and undergo poly-ingression in a ventral direction. These poly-ingressing cells contribute to forming the hypoblast, the second layer of blastoderm. Thus, blastoderms are undergoing continuous cytoarchitectural changes during development, which result in morphological remodeling. We have previously shown that blastoderms that diapause for a prolonged time at 18 °C undergo overt morphological changes, together with an increase in tissue size, as compared to embryos kept at 12 °C; yet, the latter have a higher mitotic index [3]. These counter-intuitive findings led us to suggest that the higher mitotic index at 12 °C manifests a mitotic arrest, rather than rapid proliferation. Moreover, we have demonstrated during diapause at 12 °C that embryos present more healthy-looking and viable cells than at 18 °C [3]. The sufficient count of healthy living cells in an appropriate cytoarchitectural organization and differentiation state is a prerequisite for embryos to SRD following diapause [3,4,9]. Nevertheless, it is yet unknown whether cell cycle regulatory mechanisms, such as cell cycle progression, play a central role in regulating the embryo’s characteristics, including cell survival and morphological remodeling during diapause. 

The cell cycle is regulated by multiple regulators at different phases and checkpoints of the cell cycle. At the G1 phase, the tumor suppressor retinoblastoma protein (pRb) interacts with the E2F transcription factor to inhibit cell cycle progression [10]. Phosphorylation of pRB releases E2F, which promotes the expression of Cyclin genes, such as CyclinD and CyclinE, which drives the cells to enter into the S phase [11,12,13]. By the late S-G2 phases, CyclinA and CyclinB expression increases and degrades during mitotic exit [13,14,15]. At the G1/S transition, CyclinE interacts with cyclin-dependent kinase 2 (CDK2), whereas during the S-G2-M phases, CyclinA interacts with CDK2/1, and CyclinB interacts with CDK1 [16]. Importantly, several checkpoint kinases, such as WEE1, CHK1, CHK2 and Rad53, have been found to inhibit CDK’s activities directly or indirectly during the G1/S/G2 phases, thereby preventing cell cycle progression [17,18,19]. For instance, at the G2/M checkpoint, the kinase WEE1 directly phosphorylates CDK1 and prevents the cell from entering mitosis [20]. At cell cycle arrest, the checkpoint kinases are also indispensable for the survival of early-stage mouse embryos, at the blastocysts stage [21]. Importantly, inhibition of checkpoint kinase activity of Wee1 in mouse embryos by addition of the Wee1 inhibitor MK1775 [22] dramatically shortens G2 phase to ~30 min, which is only 25% of the expected length of the G2 phase [23]. This accelerated mitotic entry is accompanied by a compromised genomic integrity in blastocyst-stage mouse embryos, as lack of Wee1 activity can lead to DNA damage, chromosomal instability and aneuploidy [24]. WEE1 plays a similar role in maintaining genomic integrity in the mammary gland and acts as a haploid tumor suppressor, since a mutant mammary gland develops tumors [25]. Crucial for maintaining genome integrity, WEE1 is a potential target for developing therapies for treating cancer in human patients [26]. However, the cell-cycle-related molecular mechanisms are not well characterized in blastulating chick embryos, particularly during diapause. Given the high evolutionary conservation of cell cycle regulators, including WEE1, the chick embryo can serve as a model organism for efficacy studies, including toxicity, genome integrity, teratogenicity and cell survival. 

Based on our recent findings, which showed that blastoderms that diapause at 12 °C or 18 °C have a different morphology and ability to undergo cell division [3], in this study we aimed at determining the possible link between cell cycle regulation, blastoderm cytoarchitecture and the ability to SRD following diapause. Using high-resolution episcopic microscopy (HREM), qualitative and quantitative analyses were performed to characterize in detail the difference in cell proliferation, poly-ingression, cell death and tissue remodeling during diapause at 12 °C or 18 °C. Moreover, a comparative RNAseq analysis was performed in the current study, revealing differential expression of various cell cycle regulatory genes between the two diapause temperatures. Interestingly, we found that the expression of the G2/M transition regulator kinase *WEE1* was significantly increased at 12 °C diapaused embryos in comparison to the 18 °C ones. Further investigation of the role of WEE1 demonstrated that during diapause at lower temperatures, cell cycle is predominantly arrested at the G2/M transition via WEE1, which is associated with better maintenance of cell viability, blastoderm cytoarchitecture and the ability to SRD. These findings are the first to uncover molecular processes that are involved in diapause phenomenon in avian.

## 2. Materials and Methods

### 2.1. Egg Collection and Isolation of Blastoderm

Freshly laid Ross (308) broiler eggs from young flock age (28–45 weeks) were collected from a commercial farm and stored at 18 °C or 12 °C up to 28 d in a cooled incubator (SN 265959, VELP SCIENTIFICA, Usmate Velate, Italy). The temperature and relative humidity of the cooled incubator was continuously monitored throughout the experiment. Following diapause, blastoderms were isolated as previously described [27]. Briefly, the eggshell was carefully cracked, the albumin was discarded, and the yolk with the embryo was gently placed in a beaker containing phosphate-buffered saline (PBS, REF-BP507/1LD, Hylabs, Rehovot, Israel). The vitelline membrane surrounding the underlying embryo was incised with scissor and gently separated from the remaining yolk. The separated vitelline membrane with the embryo was transferred to a Petri dish containing PBS. The embryo was then cleaned by removing the adherent yolk from the embryo surface by gently washing with drops of PBS. The isolated blastoderms were either placed in RNA save solution (Cat no. 01-891-1A, Biological Industries, Beit Haemek, Israel) for RNA stabilization or fixed in 4% paraformaldehyde (PFA) in PBS. 

### 2.2. HREM Imaging

Embryos were fixed overnight in 4% PFA, washed with PBS and dehydrated in a series of methanol concentrations (25%, 50%, 75% and 100%, each for 20 min). Following dehydration, the embryos were impregnated with 50% methanol and 50% JB4 dye mix solution, overnight, on a rotating shaker at 4 °C. JB4 dye mix contains solution A (Cat no.-14270-01, Electron Microscopy Sciences, Hatfield, PA, USA), 12.5 mg/mL catalyst (Cat no.-14270-06, Electron Microscopy Sciences, PA, USA), 2.75 mg/mL Eosin B (Cat no.-861006-10G, Sigma-ALdrich, St. Louis, MO, USA) and 0.5 mg/mL Acridine orange (Cat no.-A6014-10G, Sigma-Aldrich, St. Louis, MO, USA), dissolved overnight at 4 °C and then filtered. 

The 50% methanol and 50% JB4 dye mix solution was replaced with 100% JB4 dye mix solution, and impregnation continued for 24 h at 4 °C. For curing the resin at mounting, 30 µL/mL of solution B (Cat no.-14270-04, Electron Microscopy Sciences, Hatfield, PA, USA) was added to the JB4 dye mix, followed by curing the resin overnight at 4 °C in a plastic embedding mold (Cat no.-15899-50, Polysciences Europe GmbH, Hirschberg an der Bergstrasse, Germany). Polymerized sample blocks were further hardened by overnight baking at 70 °C.

Cooled hardened sample blocks were mounted in the HREM machine (Serial no.-007, Indigo scientific, Baldock, UK), and block surface images were serially taken every 2.76 µm section throughout the entire sample in 2700 × 1800 image pixel resolution. Depending on the experimental design, selected plastic sections obtained during HREM sectioning were preserved by placing them on DDW drops on histological slides and letting them dry at room temperature. Images of the plastic section were captured using a microscope (Serial no.-469580, model—DM2000LED, Leica, Wetzlar, Germany). Image of reference 1000 µm graticule (EMS, PA, USA) was taken for each block sectioned to allow for accurate size measurements. 

All the images acquired from HREM imaging were processed, 3D reconstructed and analyzed as previously described [3,27,28] using Photoshop (Adobe Photoshop CS. (2004), Peachpit Press: Berkeley, CA, USA), ImageJ software [29,30,31] and Amira software (FEI, Amira 5.4.3., Hillsboro, OR, USA) for 3D modeling of blastoderm morphology. Briefly, the HREM-acquired serial images were inverted, processed and converted to 8 bit using Photoshop (Adobe Photoshop CS. (2004), Peachpit Press: Berkeley, CA, USA), and serial images were stacked, rotated and cropped using ImageJ software (version 1.8.0_172) [29,30,31]. The stacked images were then accessed for 3D reconstruction using Amira software. A specific region of embryos was selected and compared by quantifying their volume using the Amira software segmentation tool. Moreover, a snapshot of 3D image with the black and light-blue checkerboard background was exported for a transparency test of diapaused and non-diapaused embryos. The exported image was analyzed using ImageJ software. Briefly, the exported image was converted to 8 bit, and the gray value and area of selected background region with or without embryo tissue was measured. The data were exported for further statistical analysis. The gray value of embryo region with checkerboard background was subtracted from the gray-value background region without the embryo tissue to determine the change in black (∆B) and light-blue (∆W) background. The change in gray value was averaged [(∆B + ∆W)/2] to determine the opaqueness of tissue and compared between groups. Three to four embryos were used for each HREM image analysis.

### 2.3. RNAseq Analysis

Embryos from four distinct storage conditions (0 d-unstored, 7 d/18 °C, 28 d/18 °C and 28 d/12 °C) were processed for RNAseq gene expression profiling. Each group consisted of 4 biological repeats, thus 16 blastoderms were isolated from eggs as previously described [27]. Isolated embryos were individually placed in Eppendorf tubes and stored in liquid nitrogen until RNA extraction using RNeasy micro kit (cat no. 74004, Qiagen, Maryland, USA) on the automated Qiacube system (Qiagen). RNAseq libraries were prepared from 16 samples using 200 ng total RNA according to the manufacturer’s procedure (cat no. E7760, NEBNext UltraII Directional RNA Library Prep Kit for Illumina, MA, USA). Subsequently, mRNAs pull-up was performed using the Magnetic Isolation Module (cat no. E7490, NEB, MA, USA). All 16 libraries were mixed into a single tube with equal molarity, and the RNAseq was performed on Illumina NextSeq500 in high-output mode for 75 cycles (cat no. FC-404-2005, Illumina, USA). Fastqc (v0.11.5) was used to assess the quality of single-end reads, and the obtained reads were then aligned to chicken reference genome and annotation file (Gallus_gallus-5.0 and Gallus_gallus.Gallus_gallus-5.0.93.gtf were downloaded from ENSEMBL) using STAR aligner (STAR_2.6.0a). Htseq (0.9.1) was used to count the number of reads per gene. The differential gene expression (DEG) analysis and bioinformatics analysis of DEGs were performed as described previously [32,33,34]. DEGs between 28 d/18 °C and 28 d/12 °C were analyzed using WebGestalt [32]. Over-representation (enrichment) analysis was used for pathway analysis with the KEGG functional database [35,36]. A heat map of the enriched gene sets was generated using clustvis web-based tool [37].

### 2.4. Real-Time PCR Analysis

Three isolated embryos were pooled for RNA extraction. Subsequently, cDNA was prepared using Promega kit (REF 017319, Promega, Madison, WI, USA). The primers were designed according to the sequence information from the NCBI database using Primer3 Input (version V. 0.4.0) software [38]. qRT-PCR was performed in a final reaction volume of 10 µL with the SYBR Green PCR Master Mix Kit (REF-4309155, Applied biosystems by Thermo Fisher Scientific, Inchinnan, UK) in the applied biosystems Real-Time PCR Detection System (SN 2720011007, Applied biosystems stepOnePlus Real-Time PCR System, Singapore), using the following program: 95 °C/20 s and 40 cycles of 95 °C/1 s and 60 °C/20 s. All reactions were performed in duplicates for each sample, and GAPDH was used as a reference gene for normalization of gene expression levels. The relative gene expression values were calculated using the 2^−∆∆ct^ method. Briefly, samples from diapaused embryos were the test samples, and the 0 d-unstored fresh embryos were used as the calibrator samples. The Ct value of the target genes (*Cyclin E1*, *Cyclin A1*, *Cyclin A2*, *Cyclin* *β2* and *WEE1*) of each sample group were normalized to that of the reference *GAPDH* gene. Then, the *GAPDH*-normalized Ct value of test sample group was again normalized with calibrator samples. Finally, the expression ratio was calculated using 2^−∆∆ct^ method. Thus, the result obtained is the fold of change in expression of the target gene in the tested sample relative to the calibrator sample. The comparison of fold change gene expression level was analyzed by one-way ANOVA statistical tool. The list of primers that were used to analyze the expression of genes is given in Supplementary Table S1. 

### 2.5. BrdU Incorporation Assay In-Ovo in Embryos

Fresh, fresh +6 h incubation for positive control, 7 d/18 °C, 7 d/12 °C, 28 d/18 °C and 28 d/12 °C stored embryos were treated with 0.2 mg/mL BrdU (REF B9285-1G, Sigma, USA) in PBS, by injecting 100 µL in volume to at least 4 embryos through a shell window. Following injection, the eggshell window was sealed using Leukoplast (REF 72668-02, BSN medical GmbH, Hamburg, Germany) and placed back to its respective condition for 6 h. The embryos were isolated, fixed in 4% PFA overnight at 4 °C and dehydrated in methanol (series of steps: 25%, 50%, 75% and 100%). Dehydrated samples were stored at −20 °C in 100% methanol. Whole mount embryo samples underwent DNA denaturation steps by incubating them in 50% formadide (CAT No 00068023G500, Bio-Lab, Jerusalem, Israel), in 4XSSC in DDW, for 2 h at 65 °C under slow agitation. Subsequently, samples were rinsed in 2XSSC for 15 min and incubated in 2N HCL at 37 °C for 30 min, followed by neutralization in 0.1 M sodium borate (pH 8.5) for 10 min. The samples were then washed 6 times, 15 min each, in TBS (0.15 M NaCl and 0.1 M Tris-HCL, pH 7.5) and blocked for 1 h with 3% Normal Bovine serum in TBS containing 3% triton-X-100 (TBST, CAS-9002-93-1, Sigma, USA). Following blocking, the embryos were incubated with rat anti-BrdU IgG2a (1:200; REF- OBT0030G, Serotec, Kidlington, UK) overnight at 4 °C, washed 3 times in TBS for 15 min each, at room temperature, rinsed once in TBST for 15 min and incubated for 2 h with F(ab)2 donkey anti-rat IgG-Cy3 antibody (1:200; REF-712-166-153, Jackson ImmunoResearch Laboratories, PA, USA). The embryos were washed in TBS stained with 4′,6-diamidino-2-phenylindole (DAPI) (1:1000, REF-D9542, Sigma, USA) for 30 min at RT. Stained embryos were mounted between two coverslips using fluorescence mounting medium (REF-9990402, Immu-mount, Thermo Scientific, MI, USA) and scanned using a confocal microscope (magnification, ×10 and ×40; Leica TCS SPE, Germany). The DAPI-stained nuclei and BrdU-positive cells were counted manually to determine the percentage of BrdU incorporating cells. 

### 2.6. Immunohistochemistry

The embryos were isolated and fixed in 4% PFA overnight, washed with PBS, permeabilized with 0.2% triton in PBS (PBST), blocked in 10% NGS in PBST at 4 °C for 3 h, and immunohistochemistry steps were carried out by incubating the samples with primary anti-pH 3 antibody (diluted 1:300 in blocking buffer, REF 05-817R, clone 63-1C-8, recombinant rabbit monoclonal antibody, Millipore, Billerica, MA, USA) overnight at 4 °C as previously described [39,40,41]. The primary antibody was washed in PBST and incubated overnight at 4 °C with secondary Alexa fluor 594 conjugated anti-rabbit IgG antibody, diluted 1:300 in blocking buffer (REF A11012, Invitrogen, Waltham, MA, USA). The embryos were washed in PBS, stained with DAPI, mounted and scanned as described above. pH 3-positive cells and DAPI-stained nuclei were counted manually to calculate the percentage of pH 3-positive cells. Moreover, the distribution of the M phase in diapaused embryos was calculated by further dividing the M phase into sub-phases, M1 to M7, based on pH 3 staining that showed chromatin condensation and chromosomal rearrangement and alignment [42,43,44,45]. At least four embryos were analyzed per experimental group. 

### 2.7. Embryo Treatment with WEE1 Inhibitor-MK1775

Firstly, 15% pluronic gel (P2443-250G, Sigma, USA) was prepared in PBS at 4 °C and mixed with MK-1775 to a final concentration of 500 nm (HY-10993, MedChemExpress LLC, Stockholm, NJ, USA) [17,22,46,47]. Through a hole in eggshell to the air sac, 250 µL of MK-1775 containing gel, or gel only as control, was injected in the area near to the embryo. The window in the eggshell was sealed using Leukoplast (REF 72668-02, BSN medical GmbH, Hamburg, Germany), and embryos were stored back at 12 °C for either 24 h or 7 d, depending on the experimental design. In particular, embryos diapaused for 6 d at 12 °C were treated with MK-1775 and stored at 12 °C for an additional 24 h, whereas fresh embryos treated with MK-1775 were stored for 7 d at 12 °C. At least 4 embryos from each group were then isolated as described above, fixed in 4% PFA for at least 24 h and accessed for immunohistochemistry for pH 3 staining and Terminal deoxynucleotidyl transferase dUTP nick end labeling (TUNEL) assay.

### 2.8. TUNEL Assay

Whole mount embryos were immunostained with anti-pH 3, followed by dehydration as described above. The embryos were cleared in xylene and processed for paraffin 7 µm sections as described before [3] using microtome (RM2035, Leica, Germany). Following deparaffinization and rehydration, the sections were immersed in 0.85% NaCl for 5 min, washed in PBS for 5 min, post fixed for 15 min in 4% PFA for 15 min and washed in PBS. The sections were treated with PBS containing 2% Triton X-100 for 5 min and washed in PBS. TUNEL staining was conducted using DeadEnd Fluorometric TUNEL detection system (REF-G3250, Promega, Madison, WI, USA). For positive control, sections were treated with 25 µL/mL DNAase I (REF-M0303S, BioLabs, Ipswich, MA, USA) in DNase I buffer (pH 7.9 40 mM Tris-HCl, 10 mM NaCl, 6 mM MgCl_2_ and 10 mM CaCl_2_) for 10 min, fixed in 4% PFA and washed in DDW. The TUNEL staining was performed according to manufacturer’s instructions of TUNEL detection kit; then, the samples were stained with DAPI, washed and mounted with coverslip and mounting media (REF-9990402, Immu-mount, Thermo Scientific, USA). The intensity of TUNEL-positive cells and pH 3-positive cells was quantified as previously described [48,49,50]. 

### 2.9. Statistical Tests

Data were analyzed using the statistical tests, *t*-test, one-way ANOVA and two-way ANOVA. Data were presented as mean ± SEM. Statistical tests were performed using JMP (John’s Macintosh Project) software (JMP, 189-2007, SAS Institute Inc., Cary, NC, USA) at significance level (*p* < 0.05).

## 3. Results

### 3.1. The Cellular Morphology of the Blastoderm during Diapause 

**Figure 1:**
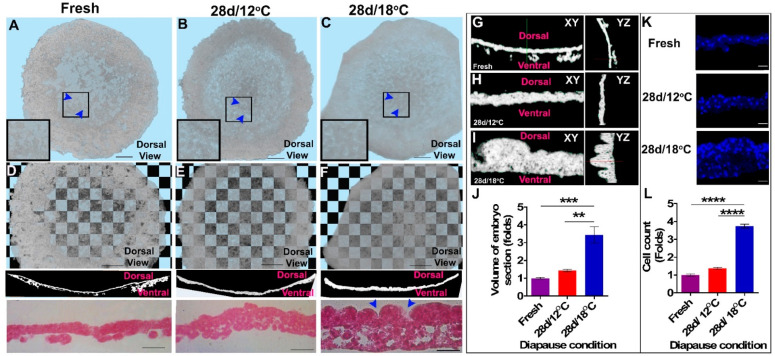
Cellular morphology of non-diapaused and prolonged diapaused (28 d/12 °C and 28 d/18 °C) blastoderm. Blastoderms were sectioned by using high-resolution episcopic microscopy (HREM) and processed by AMIRA software to obtain three-dimensional (**D**) images. (**A**–**C**) Dorsal views of blastoderms are shown. (**A**) Fresh, (**B**) 28 d/12 °C, (**C**) 28 d/18 °C. Tissue remodeling in terms of recess formation is absent in 28 d/12 °C, while it is present in 28 d/18 °C embryos (arrowhead). Big rectangular insets in (**A**–**C**) mark the magnified regions of the small rectangle. Blue arrowheads mark the regions of cytoarchitecture changes between embryos. Bar size 500 µm. (**D**–**F**) Checkerboard transparency test of fresh (**D,** upper panel, bar size 500 µm) and prolonged diapaused blastoderms (28 d/12 °C (**E**) and 28 d/18 °C (**F**), upper panel, bar size 500 µm), their corresponding HREM sections (**D**,**F**, middle panel), and plastic sections (**D**,**F**, lower panel, bar size 100 µm). Plastic section of 28 d/18 °C embryos reveals that epiblast contract to form recess (**C**, blue arrowhead) and the ventral poly-ingressing cells contribute to thickening of dorsal epiblast layer (**F**, lower panel). (**G**–**I**) XY and YZ planar views of fresh (**G**) and prolonged diapaused embryos (28 d/12 °C, (**H**), and 28 d/18 °C, (**I**)) showing their dorsal and ventral layers. (**J**) Volumetric analysis of fresh and prolonged diapaused embryos. Embryos have bigger tissue volume following prolonged diapause for 28 d at 18 °C than fresh and 28 d/12 °C (one-way ANOVA; ** *p* = 0.0021; *** *p* = 0.0007). (**K**) Embryos were sectioned in 7 micron thickness and stained with DAPI. Tissue sections were imaged using confocal laser microscopy to view nuclear staining of blastoderms. Bar size 20 µm. (**L**) Cell count analysis. Embryos consist of a greater number of cells following diapause at 28 d/18 °C, compared with fresh and 28 d/12 °C (one-way ANOVA; **** *p* < 0.0001).

**Figure 2:**
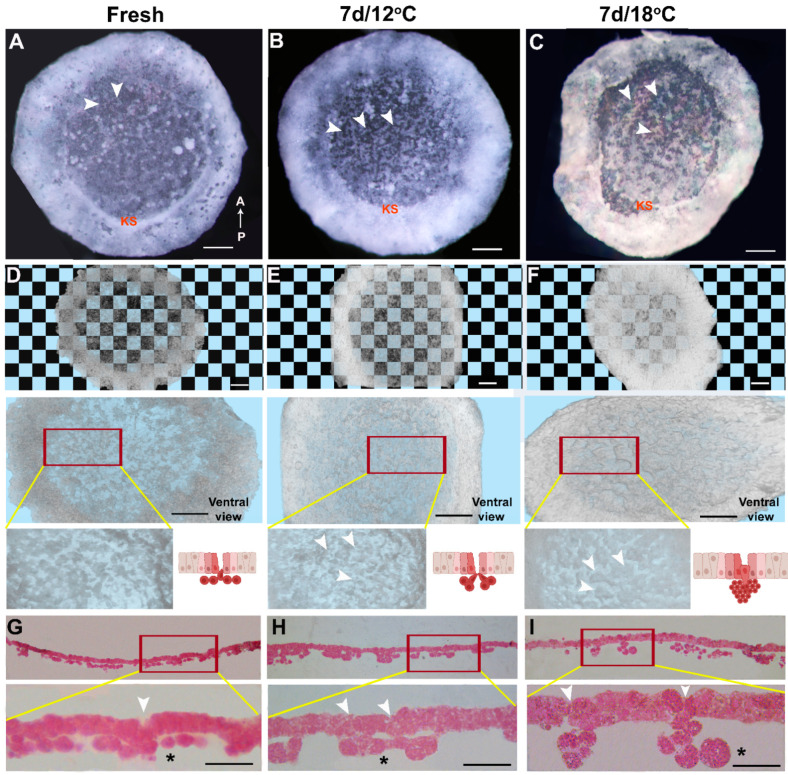
Cellular morphology of blastoderm reveals cytoarchitectural changes already within 7 d of embryo diapause at higher temperature. (**A**) Fresh. (**B**) 7 d/12 °C. (**C**) 7 d/18 °C. Poly-ingressing cells are present in anterior region (arrowhead), and Koller’s sickle (KS) marks the posterior region of blastoderm. Bar size 500 µm (**D**–**F**) HREM images showing dorsal view of blastoderms that underwent checkerboard transparency test. (**D**) Fresh, (**E**) 7 d/12 °C, (**F**) 7 d/18 °C. Embryos are more opaque in 28 d/18 °C condition than in fresh and 28 d/12 °C. Bar size 500 µm. Ventral view of same blastoderm (**D**–**F**, middle panel, bar size 500 µm) and their poly-ingressing cells are shown in high-magnification HREM images (**D**–**F**, lower panel, arrowhead) and represented in schematic diagram (**D**–**F**, lower panel). Cells that undergo poly-ingression dorso-ventrally from the epiblast region have directional cell movement, as is observed in fresh (**D**, lower panel) and 7 d/12 °C embryos (**E**, lower panel). While already following 7 d of diapause at 18 °C, there is disruption in directional cell movement (**F**, lower panel). These differences can be appreciated in plastic sections of the blastoderm obtained following HREM imaging (**G**–**I**, mag-×20). (**G**–**I**, lower panel) High-magnification images of plastic section (×40). Plastic sections showing cellular changes in blastoderms following 7 d of diapause. The polyingressing cells and clusters are marked with asterisks, lower panels. Mostly arrange as a single monolayer in the fresh and 7 d/12 °C groups, the polyingressing cells form large clusters in the 7 d/18 °C group. Bar size 100 µm.

The cytoarchitectural structure of prolonged diapaused embryos was investigated by three-dimensional (3D) HREM imaging [3,28,51,52]. To qualitatively analyze the overall thickness of the blastoderms, the 3D reconstructed images (Figure 1A–C) were subjected to a transparency test against a checkerboard background (Figure 1D–F, upper panel). The transparency test showed that embryos diapaused for 28 d at 18 °C (Figure 1F, upper panel) are more opaque than those diapaused at 12 °C or than freshly laid embryos (Figure 1D,E, upper panels). Image-analysis-based quantification of the transparency revealed a significant increase in the opacity of embryos following diapause (Figure S1A). To confirm that the transparency test accounts for the difference in embryonic thickness, the HREM images (Figure 1D–F, middle panel) and plastic sections (Figure 1D–F, lower panel) of the blastodermal tissue were analyzed at a high resolution. Images acquired from the plastic sections showed that the poly-ingressing cells in fresh embryos migrated ventrally (Figure 1D, lower panel), whereas embryos diapaused for 28 d at 18 °C were thicker, with notable recesses at the dorsal side of the epiblast (Figure 1F, lower panel, blue arrowhead; also shown in Figure S2). Notably, such recesses were not apparent in embryos diapaused at 12 °C (Figure 2E, lower panel).

In addition, using multi planar view (MPV) and segmentation tool enabled the visualization of the XY and YZ planes of the blastoderm (Figure 1G–I) and volume quantification (Figure 1J). The quantification of the unit volume of the epiblast region by segmentation tool showed a significant increase in tissue volume of blastoderms that diapaused at 18 °C compared to 12 °C or to non-diapaused embryos (Figure 1J, ** *p* = 0.0021, *** *p* = 0.0007, respectively). To investigate whether the thickening of blastoderm tissues after prolonged diapause at 18 °C was due to cell hypertrophy or to cell proliferation, we further sectioned the embryos and stained their nuclei with DAPI (Figure 1K) to allow for nuclei count. Quantifying cell number per unit area (Figure 1L), our results showed a significant increase in cell number in the 28 d/18 °C group compared with the fresh and 28 d/12 °C groups (Figure 1L, **** *p* < 0.0001), suggesting that cell proliferation majorly accounts for the thicker tissue in embryos diapaused at 18 °C.

To identify at which time point the cellular changes are initiated, we compared freshly laid embryos (Figure 2A,D,G) with embryos diapaused for 7 d at 12 °C (Figure 2B,E,H) or 18 °C (Figure 2C,F,I), using HREM sectioning. The 3D images were used for the transparency test (Figure 2D–F, upper panel; Figure S1B), which demonstrated that already after 7 d at 18 °C, the embryos are more opaque compared with the fresh and 12 °C groups. In addition, a higher-magnification view of the ventral surface (Figure 2D–F, middle and lower panels) revealed that the poly-ingressing cells in the 7 d/18 °C group tend to form large clusters, rather than forming a linear thin layer of the hypoblast. This was further validated by corresponding images from the plastic tissue sections obtained during the HREM process (Figure 2G–I). These findings highlight the aggregation of poly-ingressing cells after a short diapause at 18 °C, which is not observed in 7 d/12 °C-embryos or in freshly laid embryos (Figure 2G–I, upper and lower panels, asterisk denotes the poly-ingressing cells).

**Figure 3:**
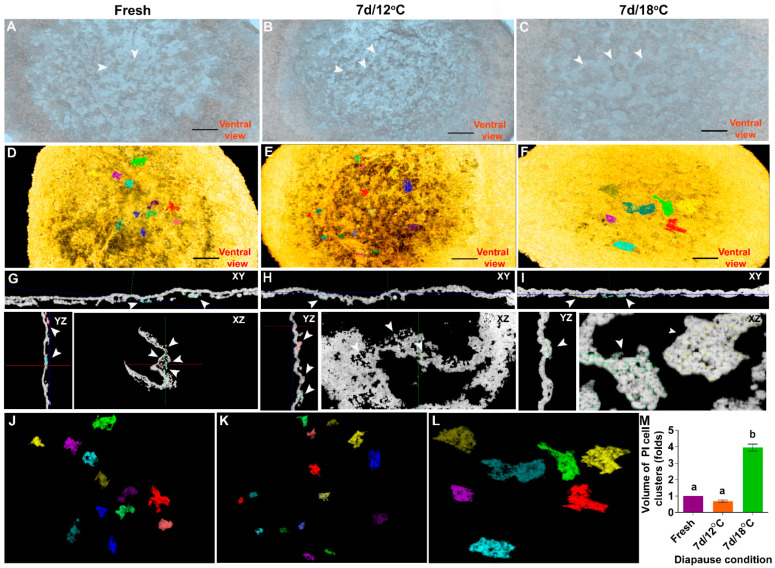
Quantification of volume of poly-ingressed cells of blastoderms. (**A**–**C**) 3D image of blastoderms showing their dorsal view. (**A**) Fresh, (**B**) 7 d/12 °C, (**C**) 7 d/18 °C. Bar size 250 µm. (**D**–**F**) Poly-ingressing cells of blastoderm represented in different colors. (**D**). Fresh, (**E**) 7 d/12 °C, (**F**) 7 d/18 °C. Bar size 250 µm. (**G**–**I**) Planar views of blastoderm showing poly-ingressing cells in different colors. (**J**–**L**) Poly-ingressing cells of blastoderm from fresh, 7 d/12 °C and 7 d/18 °C condition. (**M**) Quantification of volume of poly-ingressing cells (relative to fresh in folds). Firstly, the serial section images of fresh, 7 d/12 °C and 7 d/18 °C embryos were inverted using Photoshop software, and then processed images were stacked together using ImageJ software. The stacked images were accessed in Amira software to generate 3D images. The segmentation tool editor allows going through all the sections of a 3D image generated. The poly-ingressing cells were selected in every three intervals of serial section using blow tool, and the selected regions were interpolated. This allows for selecting each serial section. Next, all image slices were selected again, which allows for viewing further the selected region. The above steps of segmentation tool editor were used to select other poly-ingressing cells by creating new materials. After several poly-ingressing cells were selected, the isosurface editor within the object pool option allows seeing all the selected poly-ingressing cells in 3D view. Next, the volume of selected poly-ingressing cells can be quantified using material statistics option. The obtained data regarding the volume of poly-ingressing cells of each embryo group were exported for statistical analysis. Volume of poly-ingressing cells is bigger at 7 d/18 °C condition, compared with fresh and 7 d/12 °C. Different connecting letters mean that the groups are significantly different (one-way ANOVA; a vs. b: *p* < 0.0001).

To quantify the differences between the poly-ingressing cell clusters between the blastoderms, the volume of cell clusters was individually measured using the 3D segmentation tool (Figure 3). This approach enables discriminating the poly-ingressing cells from the entire overlying blastodermal cells. This was performed by fixing either freshly laid embryos (Figure 3A,D,G,J) or those diapaused for 7 d at 12 °C (Figure 3B,E,H,K) and at 18 °C (Figure 3C,F,I,L) and sectioning them using HREM. To precisely determine the boundaries of the poly-ingressing cell clusters, multi-planar view was used (Figure 3G–I), and the volume of isosurface models (Figure 3J–L) was calculated using Amira software (Figure 3M). The results show that, while the size of the poly-ingressing cell clusters in freshly laid embryos and embryos diapaused for 7 d at 12 °C are similar (*p* = 0.1152), poly-ingressing clusters in embryos diapaused for 7 d at 18 °C are significantly larger (*p* < 0.0001). Combined with the transparency test (Figure S1B), these results indicate that smaller poly-ingressing cell clusters tend to increase the opacity of embryos in 7 d/12 °C group, while bigger poly-ingressing cell clusters contribute to increased opacity in the 7 d/18 °C group. Thus, our results suggest that cellular changes in embryos are initiated already in the first week of diapause and develop into a thicker epiblast with notable recesses and higher cell count after a longer diapause, particularly at 18 °C.

Altogether, these results indicate that diapausing at 18 °C leads to cytoarchitectural changes in the blastoderms that are not found in non-diapaused embryos or in ones diapaused at 12 °C. This difference, which is already apparent after 7 d, involves a significant increase in cell cluster formation, thickening of the blastoderm, formation of recesses in the epiblast and increased cell count.

### 3.2. Transcriptome Profiling Reveals Distinct Regulation in Cell Cycle in Diapaused Embryos at 18 °C and 12 °C

**Figure 4:**
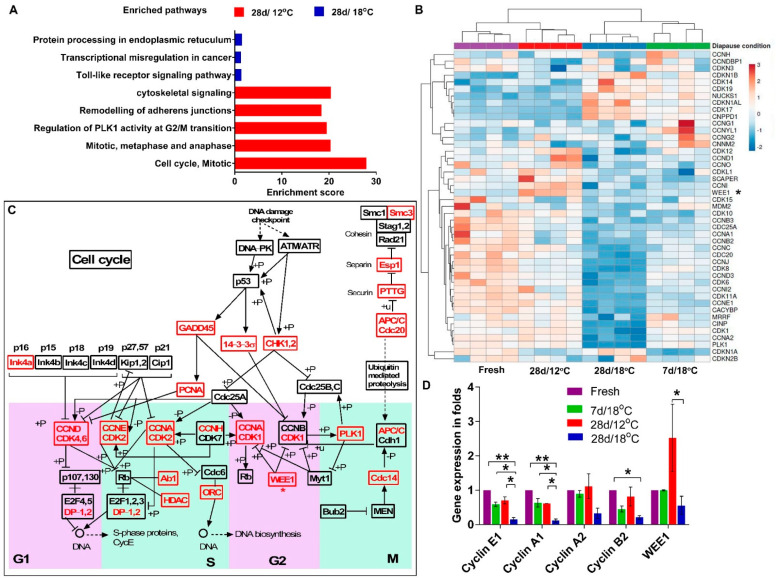
RNAseq analysis and validation. RNAseq analysis was conducted in four groups (Fresh/Control, 7 d/18 °C, 28 d/12 °C and 28 d/18 °C) with four replicates each. (**A**) Pathway enrichment analysis between 28 d/12 °C and 28 d/12 °C group. (**B**) Heat map of gene sets of embryos in different diapause conditions. (**C**) A schematic representation of enriched cell cycle pathway, as obtained by KEGG pathway analysis of DEGs between 28 d/12 °C and 28 d/12 °C group. The enriched gene sets are marked in red. An expanded scheme of enriched pathway is showed in Figure S3. (**D**) The expression of key genes involved in cell cycle was validated by using real−time PCR analysis. The expression level of target genes of each sample was normalized to that of GAPDH. The gene expression level (folds) is the GAPDH−normalized expression of target genes of 7 d/18 °C, 28 d/12 °C and 28 d/18 °C group relative to that of fresh (control) group. (one−way ANOVA; * *p* = 0.02; ** *p* = 0.0096).

The major cytoarchitecture changes and the increased cell count found in embryos diapaused at 18 °C suggest that molecular mechanisms regulating the cell cycle may be involved in these phenomena. To identify these mechanisms, RNAseq analysis was conducted in four groups of embryos: (i) freshly laid embryos, which serve as a reference control, (ii) embryos diapaused for 7 d at 18 °C (7 d/18 °C), (iii) embryos diapaused for 28 d at 18 °C (28 d/18 °C) and (iv) embryos diapaused for 28 d at 12 °C (28 d/12 °C). The differences in molecular pathways between 28 d/12 °C and 28 d/18 °C groups were analyzed using enriched Kyoto Encyclopedia of Genes and Genomes (KEGG) pathways (Figure 4A). This revealed the enrichment of pathways associated to cytoskeletal signaling, remodeling of the adherent junctions, regulation of PLK1 activity at the G2/M transition and cell cycle regulation pathways at 28 d/12 °C, whereas protein processing in the endoplasmic reticulum, transcriptional misregulation in cancer and toll-like receptor signaling pathway were enriched at 28 d/18 °C. Further analysis of the enriched gene sets showed that genes involved in cell cycle progression, including *Cyclins* and *CDKs*, were expressed in the fresh, 7 d/18 °C and 28 d/12 °C groups, while in the 28 d/18 °C group, these genes were downregulated (Figure 4B). Notably, *WEE1*, the G2/M transition regulator kinase, was found to be upregulated in 28 d/12 °C (Figure 4B, see asterisk). Moreover, the KEGG pathway analysis revealed enrichment of cell cycle pathway and linkage between genes during each cell cycle transition phase (Figure 4C, see also Figure S3), further indicating differences in the activity of cell-cycle-related genes between embryos at different diapause conditions.

**Figure 5:**
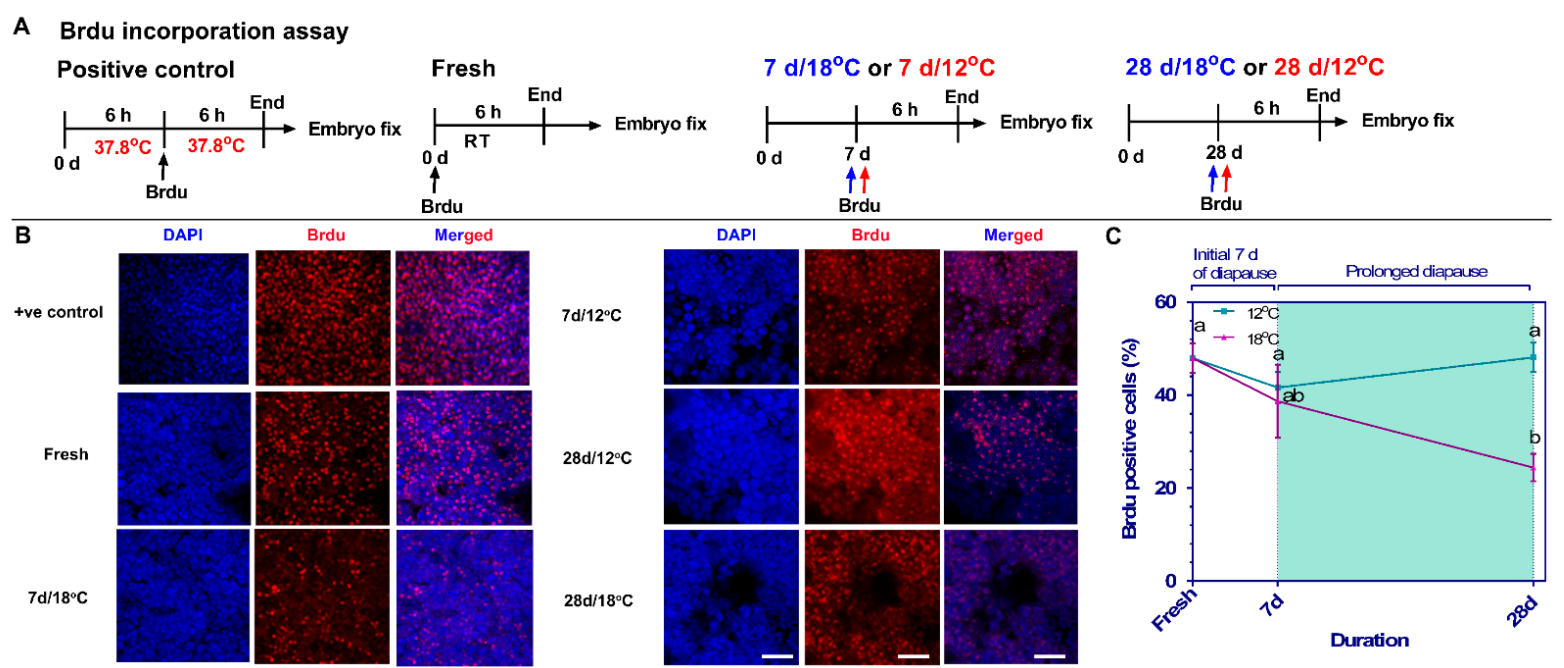
BrdU incorporation assay in blastoderms at different diapause conditions. (**A**) Experimental design. Positive control group consists of 6 h incubated embryos that were injected with BrdU for 6 h more. Likewise, fresh, 7 d/12 °C, 7 d/18 °C, 28 d/12 °C and 28 d/18 °C groups were injected with BrdU during 0 d, 7 d and 28 d of diapause for 6 h, respectively. Following treatment, embryos were isolated, fixed, immunostained with anti-BrdU antibody and accessed for confocal microscopy. (**B**) BrdU incorporation assay in positive control, fresh, 7 d/18 °C, 7 d/12 °C, 28 d/12 °C and 28 d/18 °C group. Bars 20 µm. (**C**) Quantification of BrdU-positive cells. Prolonged diapaused embryonic cells still incorporate BrdU at lower temperature, which is significantly higher than that of cells diapaused at higher temperature (*p* = 0.0159). Different connecting letters mean that the compared groups are significantly different to each other (one-way ANOVA; a vs. b: *p* = 0.001).

To examine and validate the expression of enriched cell-cycle-related gene sets in the RNAseq data, real-time PCR analysis was conducted (Figure 4D). Expression levels of *Cyclin E1 (CCNE1)*, *Cyclin A1/2 (CCNA1/2)* and *Cyclin B2 (CCNB2)* were found be downregulated in the 28 d/18 °C group compared to the other groups (* *p* = 0.02, ** *p* = 0.0096), while *WEE1* expression was upregulated in the 28 d/12 °C group (* *p* = 0.0485 compared to 28 d/18 °C group). These results, which confirmed the RNAseq data, indicated a deceleration in cell cycling following 28 d of diapause at 18 °C. However, the morphological changes observed in the 18 °C group prompted us to further determine whether there was a shift in cell cycling from proliferation to deceleration state in the 18 °C group and whether the maintained cytoarchitecture at 12 °C was associated with safeguarding of cell cycle progression through higher *WEE1* expression. As a first step to experimentally examine the cell cycle state of embryonic cells, BrdU incorporation assay was performed in order to quantify the number of cells in the S phase of the cell cycle. Different experimental groups were treated with BrdU: 1). As a positive control, fresh embryos were incubated at 37.8 °C for 6 h, treated with BrdU and further incubated for 6 h before fixation; 2). As a starting reference point that refers to the embryonic state before diapause, fresh embryos were treated with BrdU for 6 h at room temperature before fixation; 3). For the 7 d time point, embryos were diapaused at 18 °C or 12 °C for 7 d, then treated with BrdU for 6 h and fixed; and 4). For the 28 d time point, embryos were diapaused at 18 °C or 12 °C for 28 d, treated with BrdU for 6 h and fixed (Figure 5A). Following fixation, embryos were stained with anti-BrdU antibody and DAPI to visualize the nuclei, which allowed us to calculate the proportion of BrdU-positive cells out of the total nuclei count (Figure 5B). Interestingly, our results showed a reduction in BrdU incorporation within embryos diapaused at 18 °C already after 7 of diapause, which became statistically significant following 28 d (*p* = 0.001), while at 12 °C, BrdU incorporation remained stable throughout the 28 d of diapause and was comparable to fresh embryos (Figure 5B,C). These results, which stand in agreement with the RNAseq data showing stable Cyclin genes expression over an extended diapause period at 12 °C (Figure 4B–D), can suggest a marked deceleration in cell cycling following extended diapause at 18 °C, as opposed to the 12 °C group and fresh group. However, the preserved cytoarchitecture and the moderate increase in cell number in prolonged diapaused embryos at 12 °C, in contrast to the higher cell count and thicker blastoderms observed at 18 °C, argues against such scenario. Hence, an alternative explanation for the results of BrdU incorporation at 12 °C is that the cell cycle might be regulated at other transition phases following the S phase, leading to cell cycle arrest at these conditions. Thus, based on RNAseq analyses (Figure 4B–D), we hypothesized that cell cycle regulation at 12 °C may be mediated by the high expression of the negative G2/M transition regulator *WEE1* that prevents the further progression of the cell cycle toward mitosis [20]. 

### 3.3. Mapping the Mitotic Phase of Diapaused Embryos Reveals an Increase in Cell Proliferation at Higher Temperature

**Figure 6:**
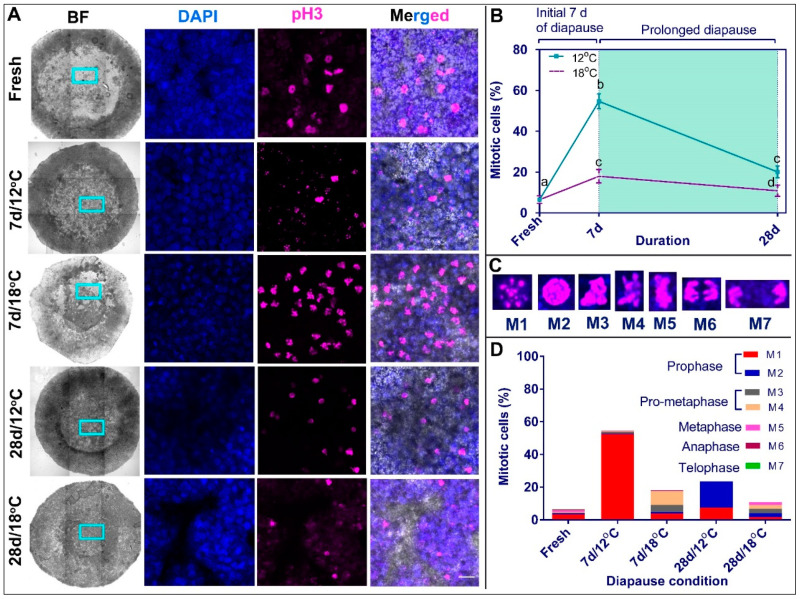
Mapping of mitotic phase of blastoderms in different diapause conditions using pH 3 immunostaining. (**A**) Confocal images showing BF, DAPI, pH 3 and merged channels of fresh, 7 d/12 °C, 7 d/18 °C, 28 d/12 °C and 28 d/18 °C groups. Bars 20 µm. (**B**) Quantification of mitotic cells. Different connecting letters mean that the compared groups are significantly different to each other (one-way ANOVA; b vs. c: *p* < 0.0001; c vs. d: *p* = 0.0028). (**C**) Sub-categorization of mitotic cells based on pH 3 staining and nuclear condensation. (**D**) Distribution of mitotic phases in blastoderms that underwent different diapause conditions (fresh, 7 d/12 °C, 7 d/18 °C, 28 d/12 °C and 28 d/18 °C).

The M phase is a complex stage of the cell cycle, involving major reorganization at the cell’s nuclei, cytoskeleton, cytoplasm and membranes [53]. The M phase is subdivided into several stages, prophase, metaphase, anaphase and telophase, which initiate with chromatin condensation and terminate with the separation into two daughter cells [42,43]. These stages can be further subdivided into more specific events: M1 and M2 stages of the prophase, which refer to the initiation and completion of chromatin condensation; M3 and M4 stages of the pro-metaphase, which refer to the initiation or near completion of chromosome’s segregation; M5 stage of the metaphase, which refers to the alignment of the chromosomes along the metaphase plate; M6 stage of the anaphase, which refers to the separation of the chromosomes into the opposite poles; and M7 stage of the telophase, which refers to the reformation of the nuclei and daughter cells [42,44,45]. As our results demonstrate changes in the proliferation state of embryos stored at different diapause temperatures (Figure 5), as well as difference in the expression levels of the G2/M regulator *WEE1* (Figure 4), here we further examined whether these findings are associated with distribution of cells at different sub-phases of the M stage of the cell cycle. Embryos diapaused at 12 °C and 18 °C for 7 and 28 d, as well as freshly laid embryos, were stained with the M phase marker pH 3 (Figure 6A) and analyzed for their mitotic phase (Figure 6B–D). The mitotic index was calculated based on the chromosomal state at M1–M7 stages (Figure 6C).

Calculating the percentage of mitotic/pH 3^+^ cells, our results show that at 12 °C, there are more pH 3^+^ mitotic cells than in fresh embryos (Figure 6A,B; fresh vs. 7 d, *p* < 0.0001; fresh vs. 28 d, *p* = 0.001) and in embryos diapaused at 18 °C (Figure 6A,B; during 7 d, *p* < 0.0001; during 28 d, *p* = 0.0028). However, the quantification of discrete M sub-phases revealed that fresh embryos consisted of cells in all mitotic phases, while embryos diapaused at 12 °C were in the prophase stage (*p* < 0.0001), which corresponds to the G2/M transition (Figure 6D). In contrast, embryos diapaused at 18 °C consisted of cells in pro-metaphase during 7 d (predominant M4 stage, *p* ≤ 0.002) and in all mitotic sub-phases during 28 d, which distributed similarly along the different stages (Figure 6D). These results suggest that the preserved cytoarchitecture of blastoderms and the higher count of BrdU+ nuclei in the 28 d/12 °C group (Figure 5) is likely due to the ability of cells to pass the S phase but arrest at the G2/M checkpoint at the prophase, the early mitosis stage (Figure 6). In contrast, in the 28 d/18 °C group, the abnormal cytoarchitectural growth may be due to continuous, yet slower, progression of the cell cycle beyond the prophase stage. 

### 3.4. Inhibition of WEE1 in Embryos Diapaused at 12 °C Results in Cell Cycle Progression beyond Prophase

**Figure 7:**
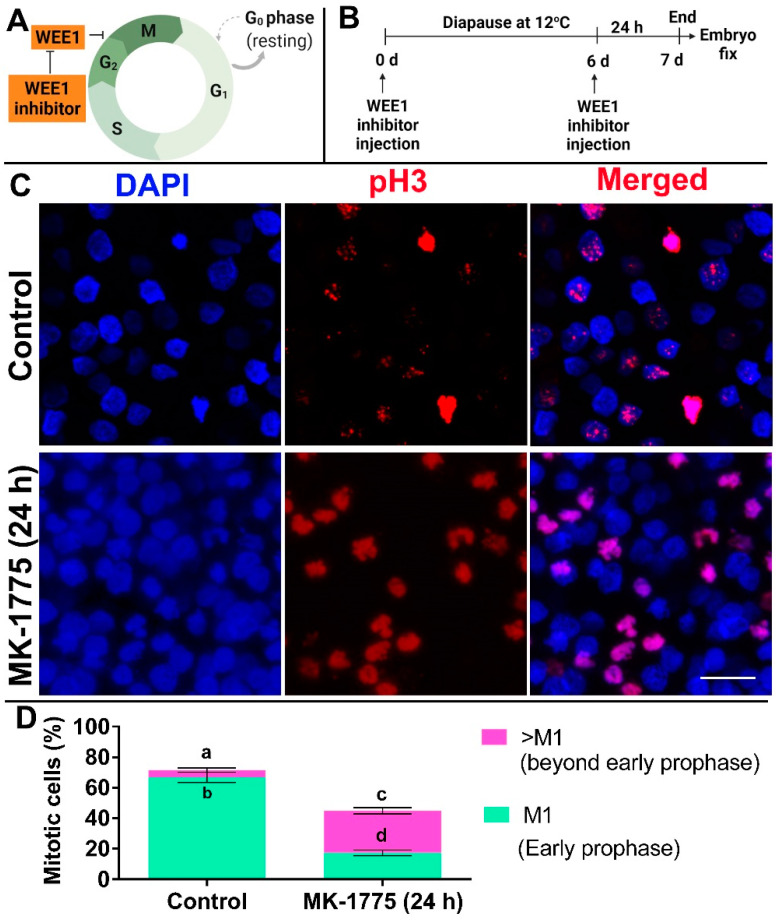
Effect of treatment of WEE1 kinase inhibitor (MK-1775) on cell cycling of blastoderm. (**A**) Schematic representation of the road map for demonstrating the effect of inhibiting WEE1 kinase activity on cell cycling. (**B**) Experimental design. Embryos were subjected to diapause for 6 d at 12 °C and, subsequently, on 6^th^ d, they were injected with WEE1 inhibitor for 24 h. PBS-treated embryos served as control. Following treatment, embryos were isolated, fixed, immunostained with anti-pH 3 antibody and accessed for confocal microscopy. (**C**) pH 3 immunostaining of blastoderms that were untreated or treated with MK-1775 for 24 h in 6 d/12 °C stored embryos. Bars 20 µm. (**D**) Quantification of mitotic cells in blastoderms without or with treatment of MK-1775 for 24 h in 6 d/12 °C group. Different connecting letters mean that the compared groups are significantly different to each other (two-way ANOVA; a vs. b: *p* = 0.0079; c vs. d: *p* = 0.011; a vs. c: *p* = 0.0079).

The above results revealed that during diapause at 12 °C, the majority of M phase cells are arrested in prophase, which correlates with the high expression of *WEE1.* Therefore, to determine the role of WEE1 in this G2/M arrest, the small molecule MK-1775, which is a highly specific and potent inhibitor of WEE1 kinase activity [46,47,54,55], was used. Embryos were diapaused at 12 °C for 6 d, treated with 500 nm MK-1775 or with PBS (as a control solution) for another 24 h, stained for pH 3 and quantified for their M1–M7 stage (Figure 7A–D). Our results show that, while in the control embryos, the majority of the mitotic cells remained at the prophase stage of the M phase (Figure 7C,D, *p* = 0.0079), in MK-1775-treated embryos, the cells were found in more advanced stages, from the pro-metaphase M3/M4 stages and onward (Figure 7D, *p* = 0.0111). These results strongly support a regulatory role of WEE1 in preventing cell cycle progression in embryos diapaused at low temperature.

**Figure 8:**
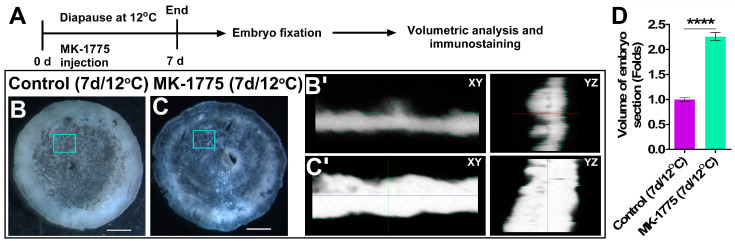
Inhibition of WEE1 kinase activity up to 7 d at 12 °C results in cellular changes in embryos. (**A**) Experimental design. Fresh embryos were treated with MK-1775 and, subsequently, they underwent diapause for 7 d at 12 °C. PBS-treated embryos under the same condition were used as control. Following treatment, embryos were isolated, fixed and subjected to volumetric analysis using HREM 3D imaging. In a separate experiment, the treated and control embryos were isolated, fixed and immunostained with anti-pH 3 antibody and accessed for confocal microscopy (Figure S4). (**B**) Control embryos. Bars 500 µm. (**C**) MK-1775-treated embryos. Bars 500 µm. While the clusters of poly-ingressing cells are visible in control embryos, MK-1775-treated embryos undergo cellular changes in terms of hypoblast progression and thickening of tissue. The inset of (**B**,**C**) is shown in (**B’**,**C’**), which represent the 3D modeling of embryo section. (**B’**) 3D images of control embryos and their visualization in XY and YZ planar views. (**C’**) 3D images of MK-1775-treated embryos and their visualization in XY and YZ planar views. (**D**) Volumetric analysis shows that MK-1775-treated embryos are twice as thick as non-treated control embryos (**** *p* < 0.0001).

To test whether the activity of WEE1 is coupled with the thinner blastoderm’s phenotype observed in the 12 °C diapausing embryos (Figure 8A), such embryos were treated with PBS (Figure 8B) or MK-1775 (Figure 8C) for 7 d and analyzed for their blastoderm cytoarchitecture using HREM sectioning (Figure 8B’,C’,D). Additional embryos were stained for pH 3 to quantify their M phase sub-distribution following 7 d of storage at 12 °C (Figure S4). Our results demonstrate that PBS-treated embryos were significantly thinner, with reduced volume (Figure 8B,B’) in comparison to the MK-1775-treated embryos, which demonstrated thicker blastoderms (Figure 8C,C’,D, *p* < 0.0001). Moreover, in control embryos, the poly-ingressing cell clusters were smaller and discreetly arranged (Figure 8C,C’, see also Figure 2B,H), while in the MK-1775-treated embryos, the poly-ingressing cells formed a continuous thick layer, covering the ventral side of the epiblast (Figure 8C,C’) resembling poly-ingressing cells of the 18 °C diapaused embryos (Figure 1 and Figure 2). Furthermore, these phenotypes were coupled with an increase in the number of pH 3-positive cells in advanced mitotic sub-phases compared to the PBS group (Figure S4, *p* < 0.0001). These results suggest that during diapause at 12 °C, the increased levels of *WEE1* arrest the cells at the G2/M, thereby inhibiting proliferation and thickening of the blastoderm, while following WEE1 inhibition with MK-1775, the cells progress with the cell cycle, which results in blastoderm thickening, similar to the phenomenon observed during diapause at 18 °C, when *WEE1* expression levels are low.

### 3.5. Increase in Staining of Cellular Death Marker, TUNEL upon WEE1 Kinase Activity Inhibition in 12 °C Diapaused Embryos 

**Figure 9:**
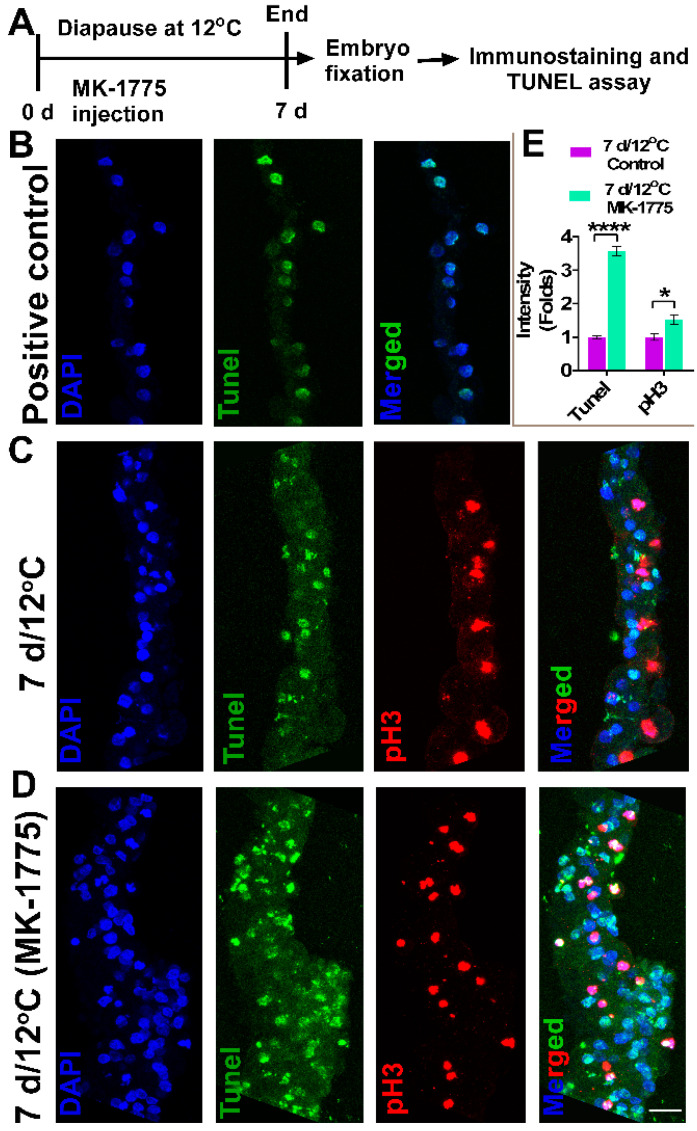
TUNEL assay in diapaused embryos. (**A**) Experimental design. Fresh embryos were treated with MK-1775 and, subsequently, they underwent diapause for 7 d at 12 °C. PBS-treated embryos under the same condition were used as control. Following treatment, embryos were isolated, fixed, immunostained with anti-pH 3 antibody, sectioned, underwent TUNEL assay in the same slide (except for positive control) and were accessed for confocal microscopy. Confocal images of all the tissue sections were acquired using the same imaging settings. (**B**) Positive control of TUNEL assay. Sections from fresh embryos were treated with DNAse to induce DNA breaks and accessed for TUNEL assay. (**C**) TUNEL assay and pH 3 immunostaining of 7 d/12 °C group without or with MK-1775 treatment (**D**). Bars 20 µm. (**E**) Quantification of fluorescence intensity of TUNEL-positive and pH 3-positive cells in blastoderms treated with or without MK-1775. *t*-test, * *p* = 0.0215; **** *p* < 0.0001.

As a G2/M checkpoint regulator, WEE1 was found to allow the extension of time spent in repairing DNA damage prior to mitosis [17]. Thus, the inability to properly repair DNA damage in cells prior to their progression into the M phase of the cell cycle promotes cell death in other contexts [56,57]. To check whether the inhibition of WEE1 kinase activity in 12 °C diapaused embryos induced cell death, such embryos were treated with MK-1775 or PBS and analyzed for apoptotic cell death using the TUNEL assay [58,59], together with their staining for pH 3 and DAPI to label the M phase stages (Figure 9A). As a positive control for the TUNEL assay, additional embryos were treated with DNAase to induce DNA damage (Figure 9B). The intensity of TUNEL staining was used to quantify the amount of apoptotic cells (Figure 9C–E) [48,49]. The results show that inhibition of the kinase activity of WEE1 not only increases the embryonic volume (Figure 8D, ****p < 0.0001) and the number of cells entering the M phase (Figure 9E, *p* = 0.0215, see also Figure S4), but it also increases the amount of TUNEL-positive cells (Figure 9C–E, *p* < 0.0001). Notably, TUNEL-positive cells were also pH 3-positive at mitotic phases, M4 and M5 (Figure 9D). These results further highlight that the G2/M arrest achieved by *WEE1* expression during diapause at 12 °C may serve as a survival mechanism for embryos diapaused at low temperatures by preventing accelerated cell proliferation and death, and thus, maintaining the cytoarchitectural integrity of the blastoderm. 

## 4. Discussion

### The Survivability of Embryonic Cells during Diapause Is Linked with Cell Cycle Regulation and Maintaining the Cytoarchitectural Structure

The freshly laid blastoderm consists of epithelial cells in the AP region that adhere to each other to form a single-layer epithelial sheet [60]. This is promoted by the expression of adhesion molecules at the apical and basolateral boundaries, such as tight junction protein and integrins [61]. Next, as the blastulation process progresses, some of the AP cells lose their integrity and undergo poly-ingression in a ventral direction [5]. Our previous study started to characterize the morphological changes that occur during diapause at different time points and temperatures, and demonstrated that at 12 °C, the embryos are more resilient to abnormal morphological remodeling and survive better when they resume their development [3]. Here, we utilized the HREM imaging strategy to fully uncover and quantify the cytoarchitectural differences of blastoderms after prolonged diapause at different temperatures, and by using RNAseq approach, demonstrated for the first time the molecular mechanisms involved in such changes in blastoderm’s cell morphology and cell volume in response to different diapause conditions.

Previously, 2D imaging strategies were mainly used to study morphology of blastoderms [5], which limited the viewing of the entire embryo in its intact state. Numerous studies have used micro-computed tomography (µCT), optical coherence tomography and scanning electron microscopy (SEM) to obtain 3D images of bone tissues, post-gastrulating chick embryos and also the chick blastoderms [62,63,64,65]; however, the techniques have certain limitation in reconstructing 3D images of small, thin and delicate tissue like the avian blastoderm. In order to obtain a 3D image of blastoderm, we previously used the HREM image analysis method [27]. This system was found advantageous over conventional blastoderm sectioning methods [5,65,66] and other 3D imaging methods [62,63,64,65] for observing and quantifying various blastodermal components, such as AO, AP, poly-ingressing cells and hypoblast [27], and also for obtaining high-resolution 3D images of bigger chick embryos at post-gastrulating stages [28], by utilizing the large data to generate high-resolution 3D images [67].

To identify the mechanisms underlying the morphological changes in blastoderms that diapause at different temperatures, RNAseq was performed, and this revealed that cell-cycle-related genes are distinctly regulated at the two temperatures tested, 12 °C or 18 °C. The KEGG pathway enrichment analysis further suggested that the G2/M transition regulatory gene *WEE1* [68] is providing a potential molecular regulator for the G2/M arrest in diapaused embryos at 12 °C, which is less active at 18 °C, leading to an abnormal progression in the cell cycle and overt changes in morphology. Previously, an increase in cell number during diapause of mouse embryos has been reported [69]. However, cell cycle arrest during diapause has also been shown in fish embryos [70]. Thus, previous studies have provided evidence that embryos in diapause can either increase the cell number or arrest the cell cycle, which is consistent with the results of this study showing either arrest of the cell cycle or increase in cell number following a small difference in temperature exposure during diapause in chick blastoderms. Cell cycle regulation by WEE1 is not only important for maintaining the cytoarchitecture of the blastoderm diapaused at 12 °C, but it may also be important during the transition from blastulation to gastrulation, because in *Xenopus*, it has been shown that the inhibition of Wee1 activity causes gastrulation defects by impairing key morphogenetic movements involved in gastrulation [71].

One of the main roles of the G2/M checkpoint is to assure that no DNA damage exists prior to cell division [17]. Consequently, the cell cycle arrest has been shown to promote cell survival in various cancer types, such as breast cancers, leukemia, melanoma and adult and pediatric brain tumors [68], and the abrogation of this arrest leads to cell death in breast cancer and human cervical cancer [56,57]. In agreement with this evidence, our study demonstrated that inhibition of WEE1 kinase activity resulted in cell progression from G2/M to mitosis, but many of these cells were TUNEL positive. Thus, the premature termination of the repair process may have increased the number of TUNEL-positive cells in 12 °C diapaused embryos. Interestingly, it has been previously shown that DNA damage is increased at low temperature in frog tadpole, fish and shrimps [72,73,74,75]. DNA damage activates the ATM/ATR DNA damage response mechanisms [76,77,78] found in tumors and cancerous cells, which in turn induces the expression of *WEE1* [79,80], resulting in cell cycle arrest at the G2/M phase [68]. Moreover, DNA of eukaryotic cells, including human cells arrested in the G2/M transition phase, has been found to be repaired by homologous recombination [81]. Thus, our result raises the possibility that G2/M arrest of blastodermal cells, as a result of an increased expression of *WEE1* at 12 °C, could be the response to DNA damage, which occurs at lower temperatures. This in turn leads to better embryonic survivability following diapause at 12 °C, as revealed by the lower number of Annexin-positive cells at lower temperature compared with higher temperature [3]. Thus, the evolutionary conserved role of *WEE1* and the similarities between the highly proliferating embryonic cells and related diseases in human patients may render the chick embryo as a valuable model organism for studying potential therapeutic approaches.

Laying of eggs is a common feature of oviparous animals, such as insects, birds, fish, reptiles and amphibians. However, the number of eggs laid varies among these organisms as part of their reproductive strategy [82,83,84,85,86]. Organisms such as insects adopt r-selected reproductive strategy and lay more eggs to ensure that at least some of them survive to the next generation [84,87,88]. Moreover, insect eggs lack the egg-protective physical structures of the avian eggshell [87,89,90,91]. Therefore, instead of egg diapause, it is advantageous to have developmental arrest into a more advanced stage to improve the reproductive performance of these organisms [92,93]. In some reptiles, numerous eggs are laid in a short time period in a single nest, thus assuring synchronous hatching and better chances for survivability [87,94]. Birds, on the other hand, are higher in the food web and have a small number of well-protected eggs, laid in days apart, therefore, embryo diapause phenomenon is necessary to synchronize the hatching and to ensure better parenting and rearing of the hatchlings. Moreover, by adopting a more K-selected reproductive strategy, avians lay a small number of eggs to reduce hatchling competition for food supply and to develop faster [88]. In addition, our study showed that higher temperature promotes cell proliferation, thereby increasing the cell count and tissue volume of the embryos. Moreover, embryos in this group consist of cells that are positive for Annexin [3]. In agreement, previous studies showed that proliferating human epithelial cells of colon, duodenum and esophagus tissues, mouse embryonic fibroblasts, mouse and human stem cells and cancer cells undergo replication stress, during which they are negative for proliferation marker but positive for cell death markers [95,96,97,98,99]. This is also the hallmark of aging phenotype [100]. Therefore, trigging replication stress has been used as a therapeutic strategy against cancer cells [101,102]. Consistent with these studies performed in different systems, the results of our study in the chick model suggest that blastodermal cells may also be subjected to replication stress after prolonged diapause at 18 °C. However, lowering the diapause temperature to 12 °C prevents this process by regulating cell-cycle-related molecular processes by upregulating *WEE1*, allowing embryos to withstand the colder temperature for a longer duration of diapause. WEE1 is an evolutionarily conserved tyrosine kinase that controls the cell cycle from yeast to humans [103,104], and our study further demonstrates the role of WEE1 during diapause in improving the avian reproduction capabilities. Involved in embryonic survivability, *WEE1* expression is dependent on minute changes in environmental temperature; thus, warmer springs may lead to increased risk of chick mortality of wild birds [105]. Conversely, an artificial setting that allows temperature control can harness the beneficial effects of the embryonic diapause phenomenon to improve hatchability in the commercial context.

In summary, this study suggests that embryonic survival at 12 °C is associated with maintaining blastoderm cytoarchitecture and cell viability as a result of cell cycle regulation by an arrest in G2/M phase mediated by WEE1. In contrast, *WEE1* expression is not upregulated in embryos diapaused at 18 °C, suggesting that the enhanced and abnormal tissue volume at this temperature is due to progression of mitosis. Altogether, our findings provide, for the first time, a missing link between cytoarchitectural changes, cell proliferation and cell survival mechanisms in diapaused embryos.

## 5. Conclusions

In conclusion, avian embryos must adapt to low temperature exposure at the very initial stage of their development, by undergoing diapause. This allows embryos to suspend their development for a long duration and still survive after exiting from it. In this study, we used HREM image analysis to quantify the morphological changes in embryos diapaused at different temperatures, 12 °C and 18 °C. In particular, we found that the cytoarchitecture of the embryos diapaused at 12 °C was better preserved than that of embryos diapaused at 18 °C. This characteristic was coupled with an arrest in the cell cycle at the G2/M stage, by upregulation of *WEE1* at 12 °C, but not at 18 °C. The inhibition of WEE1 kinase activity in embryos in diapause at 12 °C resulted in increased tissue size and higher staining for cell death marker, similar to embryos diapaused at 18 °C. By combining HREM imaging technology with RNAseq and molecular manipulation, this revealed a new role of WEE1 in safeguarding mitotic entry to regulate cytoarchitecture and cell survival in chick embryos during diapause.

## Data Availability

High throughput RNA-Seq data in FASTQ format have been deposited in the Dryad Digital Repository under DOI: https://doi.org/10.5061/dryad.pvmcvdnnc.

## Supplementary Materials

The following supporting information can be downloaded at: https://www.mdpi.com/article/10.3390/biomedicines10040779/s1, Figure S1: Transparency test of embryos; Figure S2: Cellular architecture of blastoderm following prolonged diapause at 18 °C; Figure S3: An expanded scheme of enriched cell cycle pathway in 28 d/12 °C group using KEGG pathway enrichment analysis; Figure S4: Investigation of the role of WEE1 in regulating the cell cycling of embryos in diapause at 12 °C by treating embryos with the WEE1 kinase inhibitor MK-1775; Table S1: Primer lists.

## References

[1] Stern C.D., Downs K.M. (2012). The hypoblast (visceral endoderm): An evo-devo perspective. Development.

[2] Fasenko G.M. (2007). Egg storage and the embryo. Poult. Sci..

[3] Pokhrel N., Cohen E.B.-T., Genin O., Ruzal M., Sela-Donenfeld D., Cinnamon Y. (2018). Effects of storage conditions on hatchability, embryonic survival and cytoarchitectural properties in broiler from young and old flocks. Poult. Sci..

[4] Pokhrel N., Sela-Donenfeld D., Cinnamon Y. (2021). The chick blastoderm during diapause, a landmark for optimization of preincubation storage conditions. Poult. Sci..

[5] Eyal-Giladi H., Kochav S. (1976). From cleavage to primitive streak formation: A complementary normal table and a new look at the first stages of the development of the chick. I. General morphology. Dev. Biol..

[6] Stern C.D., Canning D.R. (1990). Origin of cells giving rise to mesoderm and endoderm in chick embryo. Nature.

[7] Karagenc L., Sandikci M. (2010). Tissue distribution of cells derived from the area opaca in heterospecific quail-chick blastodermal chimeras. J. Anat..

[8] George-Weinstein M., Gerhart J., Reed R., Flynn J., Callihan B., Mattiacci M., Miehle C., Foti G., Lash J.W., Weintraub H. (1996). Skeletal Myogenesis: The Preferred Pathway of Chick Embryo Epiblast Cells in Vitro. Dev. Biol..

[9] Ko M.H., Hwang Y.S., Rim J.S., Han H.J., Han J.Y. (2017). Avian blastoderm dormancy arrests cells in G2 and suppresses apoptosis. FASEB J..

[10] Dyson N. (1998). The regulation of E2F by pRB-family proteins. Genes Dev..

[11] Lasorella A., Iavarone A., Israel M.A. (1996). Id2 specifically alters regulation of the cell cycle by tumor suppressor proteins. Mol. Cell. Biol..

[12] Ma J., Zeng S., Zhang Y., Deng G., Qu Y., Guo C., Yin L., Han Y., Shen H. (2017). BMP4 enhances hepatocellular carcinoma proliferation by promoting cell cycle progression via ID2/CDKN1B signaling. Mol. Carcinog..

[13] Bertoli C., Skotheim J.M., De Bruin R.A.M. (2013). Control of cell cycle transcription during G1 and S phases. Nat. Rev. Mol. Cell Biol..

[14] Yam C.H., Fung T.K., Poon R.Y.C. (2002). Cyclin A in cell cycle control and cancer. Cell. Mol. Life Sci..

[15] Huang Y., Sramkoski R.M., Jacobberger J.W. (2013). The kinetics of G2 and M transitions regulated by B cyclins. PLoS ONE.

[16] Whittaker S.R., Mallinger A., Workman P., Clarke P.A. (2017). Inhibitors of cyclin-dependent kinases as cancer therapeutics. Pharmacol. Ther..

[17] Do K., Doroshow J.H., Kummar S. (2013). Wee1 kinase as a target for cancer therapy. Cell Cycle.

[18] Collins I., Garrett M.D. (2005). Targeting the cell division cycle in cancer: CDK and cell cycle checkpoint kinase inhibitors. Curr. Opin. Pharmacol..

[19] Zegerman P., Diffley J.F.X. (2010). Checkpoint-dependent inhibition of DNA replication initiation by Sld3 and Dbf4 phosphorylation. Nature.

[20] Elbæk C.R., Petrosius V., Sørensen C.S. (2020). WEE1 kinase limits CDK activities to safeguard DNA replication and mitotic entry. Mutat. Res. Fundam. Mol. Mech. Mutagen..

[21] Takai H., Tominaga K., Motoyama N., Minamishima Y.A., Nagahama H., Tsukiyama T., Ikeda K., Nakayama K., Nakanishi M., Nakayama K.I. (2000). Aberrant cell cycle checkpoint function and early embryonic death in Chk1^(−/−)^ mice. Genes Dev..

[22] Hirai H., Iwasawa Y., Okada M., Arai T., Nishibata T., Kobayashi M., Kimura T., Kaneko N., Ohtani J., Yamanaka K. (2009). Small-molecule inhibition of Wee1 kinase by MK-1775 selectively sensitizes p53-deficient tumor cells to DNA-damaging agents. Mol. Cancer Ther..

[23] Strauss B., Harrison A., Coelho P.A., Yata K., Zernicka-Goetz M., Pines J. (2018). Cyclin B1 is essential for mitosis in mouse embryos, and its nuclear export sets the time for mitosis. J. Cell Biol..

[24] Tominaga Y., Li C., Wang R.H., Deng C.X. (2006). Murine Wee1 plays a critical role in cell cycle regulation and pre-implantation stages of embryonic development. Int. J. Biol. Sci..

[25] Vassilopoulos A., Tominaga Y., Kim H.-S., Lahusen T., Li B., Yu H., Gius D., Deng C.-X. (2015). WEE1 murine deficiency induces hyper-activation of APC/C and results in genomic instability and carcinogenesis. Oncogene.

[26] Esposito F., Giuffrida R., Raciti G., Puglisi C., Forte S. (2021). Wee1 kinase: A potential target to overcome tumor resistance to therapy. Int. J. Mol. Sci..

[27] Pokhrel N., Cohen E.B., Genin O., Cinnamon Y. (2017). Cellular and morphological characterization of blastoderms from freshly laid broiler eggs. Poult. Sci..

[28] Kohl A., Golan N., Cinnamon Y., Genin O., Chefetz B., Sela-Donenfeld D. (2019). A proof of concept study demonstrating that environmental levels of carbamazepine impair early stages of chick embryonic development. Environ. Int..

[29] Schindelin J., Arganda-Carrera I., Frise E., Verena K., Mark L., Tobias P., Stephan P., Curtis R., Stephan S., Benjamin S. (2009). Fiji—An Open platform for biological image analysis. Nat. Methods.

[30] Rueden C.T., Schindelin J., Hiner M.C., DeZonia B.E., Walter A.E., Arena E.T., Eliceiri K.W. (2017). ImageJ2: ImageJ for the next generation of scientific image data. BMC Bioinform..

[31] Schneider C.A., Rasband W.S., Eliceiri K.W. (2012). NIH Image to ImageJ: 25 years of image analysis. Nat. Methods.

[32] Liao Y., Wang J., Jaehnig E.J., Shi Z., Zhang B. (2019). WebGestalt 2019: Gene set analysis toolkit with revamped UIs and APIs. Nucleic Acids Res..

[33] Love M.I., Huber W., Anders S. (2014). Moderated estimation of fold change and dispersion for RNA-seq data with DESeq2. Genome Biol..

[34] Wan Y., Jin S., Ma C., Wang Z., Fang Q., Jiang R. (2017). RNA-Seq reveals seven promising candidate genes affecting the proportion of thick egg albumen in layer-type chickens. Sci. Rep..

[35] Yi Y., Fang Y., Wu K., Liu Y., Zhang W. (1999). Comprehensive gene and pathway analysis of cervical cancer progression. Oncol. Lett..

[36] Kanehisa M. (2019). Toward understanding the origin and evolution of cellular organisms. Protein Sci..

[37] Metsalu T., Vilo J. (2015). ClustVis: A web tool for visualizing clustering of multivariate data using Principal Component Analysis and heatmap. Nucleic Acids Res..

[38] Koressaar T., Remm M. (2007). Enhancements and modifications of primer design program Primer3. Bioinformatics.

[39] Lunn J.S., Fishwick K.J., Halley P.A., Storey K.G. (2007). A spatial and temporal map of FGF/Erk1/2 activity and response repertoires in the early chick embryo. Dev. Biol..

[40] Peretz Y., Kohl A., Slutsky N., Komlos M., Varshavsky S., Sela-Donenfeld D. (2018). Neural stem cells deriving from chick embryonic hindbrain recapitulate hindbrain development in culture. Sci. Rep..

[41] Kalev-Altman R., Hanael E., Zelinger E., Blum M., Monsonego-Ornan E., Sela-Donenfeld D. (2020). Conserved role of matrix metalloproteases 2 and 9 in promoting the migration of neural crest cells in avian and mammalian embryos. FASEB J..

[42] Slattery S.D., Newberg J.Y., Szafran A.T., Hall R.M., Brinkley B.R., Mancini M.A. (2012). A framework for image-based classification of mitotic cells in asynchronous populations. Assay Drug Dev. Technol..

[43] Walczak C.E., Cai S., Khodjakov A. (2010). Mechanisms of chromosome behaviour during mitosis. Nat. Rev. Mol. Cell Biol..

[44] Baudoin N.C., Cimini D. (2018). A guide to classifying mitotic stages and mitotic defects in fixed cells. Chromosoma.

[45] Mori S., Bernardi R., Laurent A., Resnati M., Crippa A., Gabrieli A., Keough R., Gonda T.J., Blasi F. (2012). Myb-Binding Protein 1A (MYBBP1A) Is Essential for Early Embryonic Development, Controls Cell Cycle and Mitosis, and Acts as a Tumor Suppressor. PLoS ONE.

[46] Hirai H., Arai T., Okada M., Nishibata T., Kobayashi M., Sakai N., Imagaki K., Ohtani J., Sakai T., Yoshizumi T. (2010). MK-1775, a small molecule Wee1 inhibitor, enhances antitumor efficacy of various DNA-damaging agents, including 5-fluorouracil. Cancer Biol. Ther..

[47] Kreahling J.M., Gemmer J.Y., Reed D., Letson D., Bui M., Altiok S. (2012). MK1775, A Selective Wee1 Inhibitor, Shows Single-Agent Antitumor Activity Against Sarcoma Cells. Mol Cancer Ther..

[48] Noiron J., Hoareau M., Colin J., Guénal I. (2021). Apoptosis quantification in tissue: Development of a semi-automatic protocol and assessment of critical steps of image processing. Biomolecules.

[49] Daniel B., DeCoster M.A. (2004). Quantification of sPLA2-induced early and late apoptosis changes in neuronal cell cultures using combined TUNEL and DAPI staining. Brain Res. Protoc..

[50] Weisinger K., Kayam G., Missulawin-Drillman T., Sela-Donenfeld D. (2010). Analysis of expression and function of FGF-MAPK signaling components in the hindbrain reveals a central role for FGF3 in the regulation of Krox20, mediated by Pea3. Dev. Biol..

[51] Weninger W.J., Geyer S.H., Mohun T.J., Rasskin-Gutman D., Matsui T., Ribeiro I., da Costa F.L., Izpisúa-Belmonte J.C., Müller G.B. (2006). High-resolution episcopic microscopy: A rapid technique for high detailed 3D analysis of gene activity in the context of tissue architecture and morphology. Anat. Embryol..

[52] Geyer S.H., Maurer-Gesek B., Reissig L.F., Weninger W.J. (2017). High-resolution episcopic microscopy (HREM)—Simple and robust protocols for processing and visualizing organic materials. J. Vis. Exp..

[53] Matthews H.K., Bertoli C., de Bruin R.A.M. (2022). Cell cycle control in cancer. Nat. Rev. Mol. Cell Biol..

[54] Zhang M., Dominguez D., Chen S., Fan J., Qin L., Long A., Li X., Zhang Y., Shi H., Zhang B. (2017). WEE1 inhibition by MK1775 as a single-agent therapy inhibits ovarian cancer viability. Oncol. Lett..

[55] Yuan M.L., Li P., Xing Z.H., Di J.M., Liu H., Yang A.K., Lin X.J., Jiang Q.W., Yang Y., Huang J.R. (2018). Inhibition of WEE1 Suppresses the Tumor Growth in Laryngeal Squamous Cell Carcinoma. Front. Pharmacol..

[56] Ha D.H., Min A., Kim S., Jang H., Kim S.H., Kim H.J., Ryu H.S., Ku J.L., Lee K.H., Im S.A. (2020). Antitumor effect of a WEE1 inhibitor and potentiation of olaparib sensitivity by DNA damage response modulation in triple-negative breast cancer. Sci. Rep..

[57] Lee Y.Y., Cho Y.J., won Shin S., Choi C., Ryu J.Y., Jeon H.K., Choi J.J., Hwang J.R., Choi C.H., Kim T.J. (2019). Anti-Tumor Effects of Wee1 Kinase Inhibitor with Radiotherapy in Human Cervical Cancer. Sci. Rep..

[58] Denton D., Kumar S. (2015). Terminal deoxynucleotidyl transferase (TdT)-mediated dutp nick-end labeling (TUNEL) for detection of apoptotic cells in Drosophila. Cold Spring Harb. Protoc..

[59] Feng X., Krogh K.A., Wu C.Y., Lin Y.W., Tsai H.C., Thayer S.A., Wei L.N. (2014). Receptor-interacting protein 140 attenuates endoplasmic reticulum stress in neurons and protects against cell death. Nat. Commun..

[60] Serrano Nájera G., Weijer C.J. (2020). Cellular processes driving gastrulation in the avian embryo. Mech. Dev..

[61] Harris T. (2012). Adherens Junctions: From Molecular Mechanisms to Tissue Development and Disease.

[62] Scott A.E., Vasilescu D.M., Seal K.A.D., Keyes S.D., Mavrogordato M.N., Hogg J.C., Sinclair I., Warner J.A., Hackett T.L., Lackie P.M. (2015). Three dimensional imaging of paraffin embedded human lung tissue samples by micro-computed tomography. PLoS ONE.

[63] Ugryumova N., Stevens-Smith J., Scutt A., Matcher S.J. (2008). Local variations in bone mineral density: A comparison of OCT versus x-ray micro-CT. Coherence Domain Opt. Methods Opt. Coherence Tomogr. Biomed. XII.

[64] Varner V.D., Voronov D.A., Taber L.A. (2010). Mechanics of head fold formation: Investigating tissue-level forces during early development. Development.

[65] Lee H.C., Lu H.C., Turmaine M., Oliveira N.M.M., Yang Y., De Almeida I., Stern C.D. (2020). Molecular anatomy of the pre-primitive-streak chick embryo. Open Biol..

[66] Eyal-Giladi H., Debby A., Harel N. (1992). The posterior section of the chick’s area pellucida and its involvement in hypoblast and primitive streak formation. Development.

[67] Geyer S.H., Tinhofer I.E., Lumenta D.B., Kamolz L.P., Branski L., Finnerty C.C., Herndon D.N., Weninger W.J. (2015). High-resolution episcopic microscopy (HREM): A useful technique for research in wound care. Ann. Anat..

[68] Matheson C.J., Backos D.S., Reigan P. (2016). Targeting WEE1 Kinase in Cancer. Trends Pharmacol. Sci..

[69] Kamemizu C., Fujimori T. (2019). Distinct dormancy progression depending on embryonic regions during mouse embryonic diapause. Biol. Reprod..

[70] Dolfi L., Ripa R., Antebi A., Valenzano D.R., Cellerino A. (2019). Cell cycle dynamics during diapause entry and exit in an annual killifish revealed by FUCCI technology. Evodevo.

[71] Murakami M.S., Moody S.A., Daar I.O., Morrison D.K. (2004). Morphogenesis during Xenopus gastrulation requires Wee1-mediated inhibition of cell proliferation. Development.

[72] Morison S.A., Cramp R.L., Alton L.A., Franklin C.E. (2020). Cooler temperatures slow the repair of DNA damage in tadpoles exposed to ultraviolet radiation: Implications for amphibian declines at high altitude. Glob. Chang. Biol..

[73] Dieser M., Battista J.R., Christner B.C. (2013). DNA double-strand break repair at −15 °C. Appl. Environ. Microbiol..

[74] Cheng C.H., Ye C.X., Guo Z.X., Wang A.L. (2017). Immune and physiological responses of pufferfish (*Takifugu obscurus*) under cold stress. Fish Shellfish Immunol..

[75] Qiu J., Wang W.N., Wang L.J., Liu Y.F., Wang A.L. (2011). Oxidative stress, DNA damage and osmolality in the Pacific white shrimp, Litopenaeus vannamei exposed to acute low temperature stress. Comp. Biochem. Physiol..

[76] Yang J., Xu Z.P., Huang Y., Hamrick H.E., Duerksen-Hughes P.J., Yu Y.N. (2004). ATM and ATR: Sensing DNA damage. World J. Gastroenterol..

[77] Smith J., Mun Tho L., Xu N., Gillespie D.A. (2010). The ATM-Chk2 and ATR-Chk1 Pathways in DNA Damage Signaling and Cancer.

[78] Maréchal A., Zou L. (2013). DNA damage sensing by the ATM and ATR kinases. Cold Spring Harb. Perspect. Biol..

[79] Gorecki L., Andrs M., Korabecny J. (2021). Clinical candidates targeting the ATR–CHK11–WEE1 axis in cancer. Cancers.

[80] Smith H.L., Southgate H., Tweddle D.A., Curtin N.J. (2020). DNA damage checkpoint kinases in cancer. Expert Rev. Mol. Med..

[81] Hustedt N., Durocher D. (2017). The control of DNA repair by the cell cycle. Nat. Cell Biol..

[82] Dixon A.F.G., Guo Y. (1993). Egg and cluster size in lady bird beetles (Coleoptera: Coccinellidae): The direct and indirect effects of aphid abundance. Eur. J. Entomol..

[83] Berrigan D. (1991). The Allometry of Egg Size and Number in Insects. Oikos.

[84] Morrongiello J.R., Bond N.R., Crook D.A., Wong B.B.M. (2012). Spatial variation in egg size and egg number reflects trade-offs and bet-hedging in a freshwater fish. J. Anim. Ecol..

[85] Christians J.K. (2002). Avian egg size: Variation within species and inflexibility within individuals. Biol. Rev..

[86] Taborsky B., Skubic E., Bruintjes R. (2007). Mothers adjust egg size to helper number in a cooperatively breeding cichlid. Behav. Ecol..

[87] Suetsugu K., Funaki S., Takahashi A., Ito K., Yokoyama T. (2018). Potential role of bird predation in the dispersal of otherwise flightless stick insects. Ecology.

[88] Cassill D.L. (2019). Extending r/K selection with a maternal risk-management model that classifies animal species into divergent natural selection categories. Sci. Rep..

[89] Goldberg J., Bresseel J., Constant J., Kneubühler B., Leubner F., Michalik P., Bradler S. (2015). Extreme convergence in egg-laying strategy across insect orders. Sci. Rep..

[90] Mansour N., Lahnsteiner F., Patzner R.A. (2009). Physiological and biochemical investigations on egg stickiness in common carp. Anim. Reprod. Sci..

[91] Mazzini M., Callaini G., Mencarelli C. (1984). A comparative analysis of the evolution of the egg envelopes and the origin of the yolk. Bolletino di Zool..

[92] Zhang C., Wei D., Shi G., Huang X., Cheng P., Liu G., Guo X., Liu L., Wang H., Miao F. (2019). Understanding the regulation of overwintering diapause molecular mechanisms in Culex pipiens pallens through comparative proteomics. Sci. Rep..

[93] Ishizaki H. (1972). Arrest of adult development in debrained pupae of the silkworm, Bombyx mori. J. Insect Physiol..

[94] Rafferty A.R., Reina R.D. (2012). Arrested embryonic development: A review of strategies to delay hatching in egg-laying reptiles. Proc. R. Soc. B Biol. Sci..

[95] Bai G., Smolka M.B., Schimenti J.C. (2016). Chronic DNA Replication Stress Reduces Replicative Lifespan of Cells by TRP53-Dependent, microRNA-Assisted MCM2-7 Downregulation. PLoS Genet..

[96] Calcinotto A., Kohli J., Zagato E., Pellegrini L., Demaria M., Alimonti A. (2019). Cellular senescence: Aging, cancer, and injury. Physiol. Rev..

[97] Tomasetti C., Poling J., Roberts N.J., London N.R., Pittman M.E., Haffner M.C., Rizzo A., Baras A., Karim B., Kim A. (2019). Cell division rates decrease with age, providing a potential explanation for the age-dependent deceleration in cancer incidence. Proc. Natl. Acad. Sci. USA..

[98] Xie X., Hiona A., Lee A.S., Cao F., Huang M., Li Z., Cherry A., Pei X., Wu J.C. (2011). Effects of long-term culture on human embryonic stem cell aging. Stem Cells Dev..

[99] Nayan M., Paul A., Chen G., Chiu R.C.J., Prakash S., Shum-Tim D. (2011). Superior therapeutic potential of young bone marrow mesenchymal stem cells by direct intramyocardial delivery in aged recipients with acutemyocardial infarction: In Vitro and in Vivo investigation. J. Tissue Eng..

[100] López-Otín C., Blasco M.A., Partridge L., Serrano M., Kroemer G. (2013). The Hallmarks of Aging. Cell.

[101] Carruthers R.D., Ahmed S.U., Ramachandran S., Strathdee K., Kurian K.M., Hedley A., Gomez-Roman N., Kalna G., Neilson M., Gilmour L. (2018). Replication stress drives constitutive activation of the DNA damage response and radioresistance in glioblastoma stem-like cells. Cancer Res..

[102] Ngoi N.Y.L., Sundararajan V., Tan D.S.P. (2020). Exploiting replicative stress in gynecological cancers as a therapeutic strategy. Int. J. Gynecol. Cancer.

[103] Pai C.C., Hsu K.F., Durley S.C., Keszthelyi A., Kearsey S.E., Rallis C., Folkes L.K., Deegan R., Wilkins S.E., Pfister S.X. (2019). An essential role for dNTP homeostasis following CDK-induced replication stress. J. Cell Sci..

[104] Heald R., McLoughlin M., McKeon F. (1993). Human wee1 maintains mitotic timing by protecting the nucleus from cytoplasmically activated cdc2 kinase. Cell.

[105] Shipley J.R., Twining C.W., Taff C.C., Vitousek M.N., Flack A., Winkler D.W. (2020). Birds advancing lay dates with warming springs face greater risk of chick mortality. Proc. Natl. Acad. Sci. USA.

