# Supplementary material for: HREM, RNAseq and Cell Cycle Analyses Reveal the Role of the G2/M-Regulatory Protein, WEE1, on the Survivability of Chicken Embryos during Diapause
Source: Biomedicines. 2022 Mar 27;10(4):779. doi: 10.3390/biomedicines10040779 (PMC9033001; doi:10.3390/biomedicines10040779)
Supplement: Supplementary file 1 [file biomedicines-10-00779-s001.zip › biomedicines-1615968-supplementary.pdf]

Supplementary Figures

HREM, RNAseq and Cell Cycle Analyses Reveal the Role of the G2/M-Regulatory Protein, WEE1, on the Survivability of Chicken Embryos during Diapause

Narayan Pokhrel [1,2], Olga Genin [1], Dalit Sela-Donenfeld [2,*] and Yuval Cinnamon [1,*]

[1] Agriculture Research Organization, The Volcani Center, Department of Poultry and Aquaculture Science, Bet Dagan 50250, Israel; narayan.pokhrel@mail.huji.ac.il (N.P.); olga.genin5654@gmail.com (O.G.)

[2] The Robert H. Smith Faculty of Agriculture, Food and Environment, Koret School of Veterinary Medicine, The Hebrew University of Jerusalem, Rehovot 7610001, Israel

* Correspondence: dalit.seladon@mail.huji.ac.il (D.S.-D.); yuval.cinnamon@mail.huji.ac.il (Y.C.)

Abstract: Avian blastoderm can enter into diapause when kept at low temperatures and successfully resume development (SRD) when re-incubated in body temperature. These abilities, which are largely affected by the temperature and duration of the diapause, are poorly understood at the cellular and molecular level. To determine how temperature affects embryonic morphology during diapause, high-resolution episcopic microscopy (HREM) analysis was utilized. While blastoderms diapausing at 12 °C for 28 days presented typical cytoarchitecture, similar to non-diapaused embryos, at 18 °C, much thicker blastoderms with higher cell number were observed. RNAseq was conducted to discover the genes underlying these phenotypes, revealing differentially expressed cell cycle regulatory genes. Among them, *WEE1*, a negative regulator of G2/M transition, was highly expressed at 12 °C compared to 18 °C. This finding suggested that cells at 12 °C are arrested at the G2/M phase, as supported by bromodeoxyuridine incorporation (BrdU) assay and phospho-histone H3 (pH 3) immunostaining. Inhibition of WEE1 during diapause at 12 °C resulted in cell cycle progression beyond the G2/M and augmented tissue volume, resembling the morphology of 18 °C-diapaused embryos. These findings suggest that diapause at low temperatures leads to *WEE1* upregulation, which arrests the cell cycle at the G2/M phase, promoting the perseverance of embryonic cytoarchitecture and future SRD. In contrast, *WEE1* is not upregulated during diapause at higher temperature, leading to continuous proliferation and maladaptive morphology associated with poor survivability. Combining HREM-based analysis with RNAseq and molecular manipulations, we present a novel mechanism that regulates the ability of diapaused avian embryos to maintain their cytoarchitecture via cell cycle arrest, which enables their SRD.

Keywords: chicken embryonic diapause; cell cycle; G2/M transition; chicken embryonic blastoderm; WEE1; high-resolution episcopic microscopy (HREM); RNAseq; mitosis

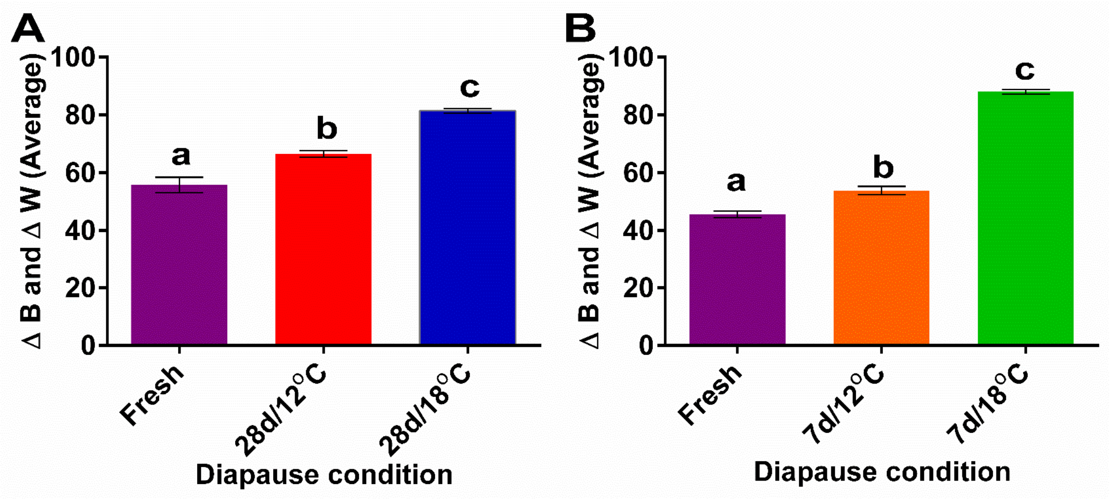

Figure S1. Transparency test of embryos. Embryos were diapaused at 12°C or 18°C for 7 or 28 day in maintained relative humidity. Fresh non-diapaused embryos served as controls. Fresh non-diapaused embryos and diapaused embryos were isolated, fixed, processed in methanol, soaked in JB4 dye and underwent HREM sectioning. Acquired images were further processed for reconstructing 3-D images using Amira software. Three-D images underwent checkerboard transparency test. The images with checkerboard background were captured and further processed in ImageJ software. Firstly, the images were converted to 8 bit and using edit/selection/specify tool, the same regions for all embryos were specified. Next, the area and mean gray value of selected region was measured using analyze and set measurement tools. The obtained data was exported for further analysis. To obtain the change in black color (ΔB), the mean gray value of embryo regions under black background was subtracted with mean gray value of black background without tissue. This value reflects the increase in opacity of tissue. Likewise, difference between mean gray value of light blue background and the tissue region under same background was measured to determine ΔW. Obtained ΔB and ΔW value was averaged [(ΔB +ΔW)/2] and compared between groups to determine the change in transparency of embryo tissue. (**A**) Transparency test of fresh, 28d/12°C and 28d/18°C embryos. (**B**) Transparency test of fresh, 7d/12°C and 7d/18°C embryos. The results showed significant increase in opacity of embryos following diapause. Statistical analysis using One-way ANOVA (In panel A; a vs. b: $p = 0.0012$; a vs. c: $p<0.0001$; b vs. c: $p<0.0001$; In panel B; a vs. b: $p<0.0001$; a vs. c: $p<0.0001$; b vs. c: $p<0.0001$).

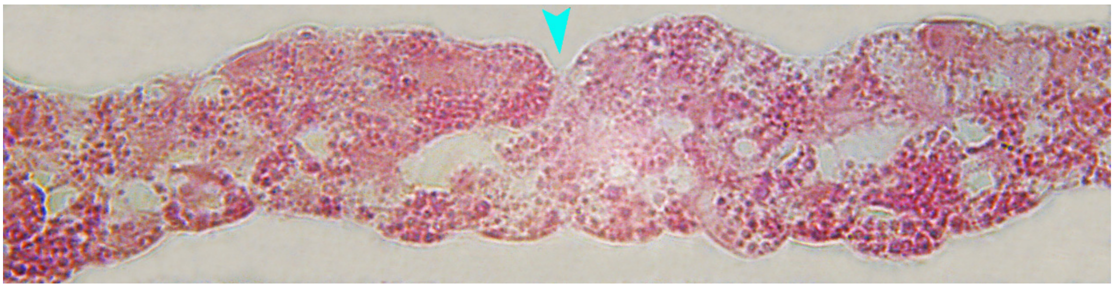

Figure S2. Cellular architecture of blastoderm following prolonged diapause at 18°C. Embryos diapaused at 18°C for 28 d were isolated, fixed in PFA for 24 h, washed with PBS, dehydrated in series of methanol (25%, 50%, 75%, and 100%, each for 20 min) and subjected to HREM analysis as described in the method section. Briefly, embryos soaked with 100% JB4 mix were embedded in plastic molds and accessed for HREM sectioning. The plastic sections were collected and placed on a slide containing a drop of DDW. This allowed the plastic tissue sections to adhere to the slide. The slide was then dried at room temperature and imaged. The

captured images were analyzed, and showed thickening of the epiblast regions and formation of recesses in embryos in diapause at 18°C for 28 d (blue arrowhead).

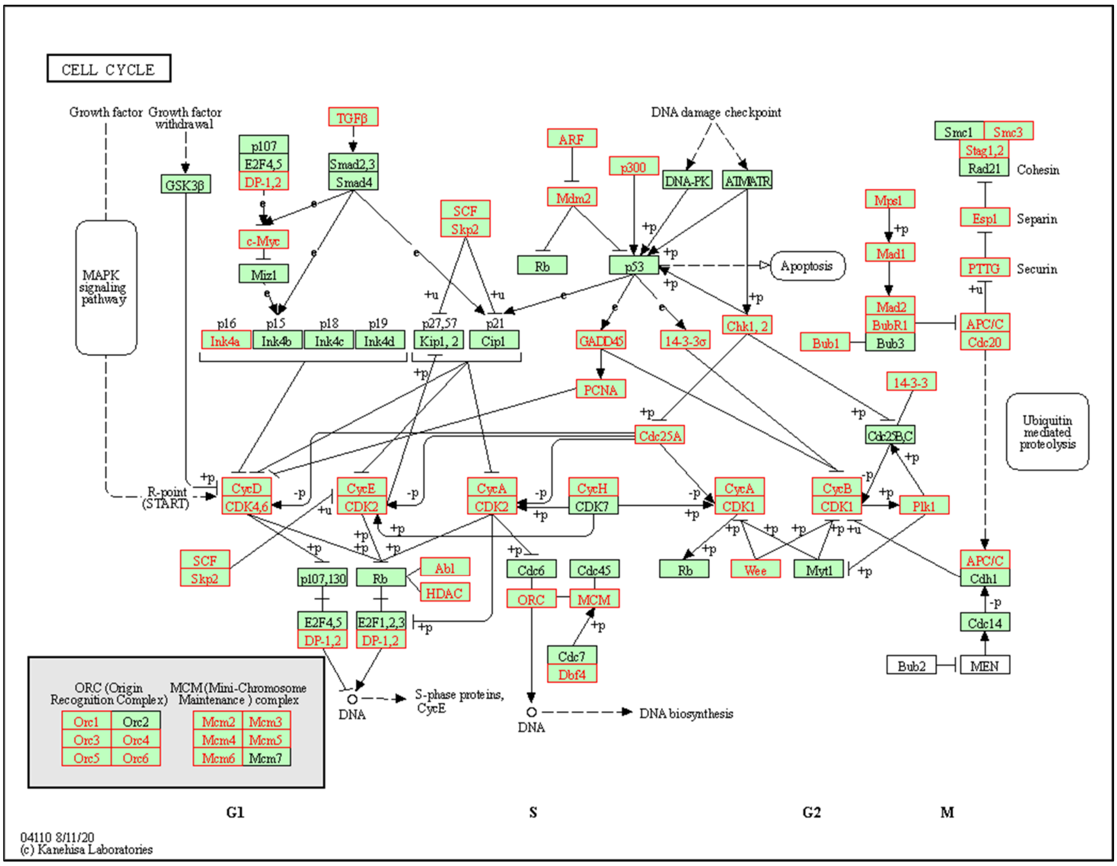

Figure S3. An expanded scheme of enriched cell cycle pathway in 28d/12°C group using KEGG pathway enrichment analysis. To uncover the molecular mechanism responsible for maintaining the cytoarchitecture of the blastoderm following 28 d of diapause at 12°C versus 18°C, RNAseq analysis was performed. Differentially expressed genes between the 28d/12°C and 28d/18°C groups were obtained and genes upregulated in each group were further examined for KEGG pathway enrichment analysis. This showed enrichment of the cell cycle pathway in the 28d/12°C group. The genes are distributed according to the cell cycle phase (G1, S, G2, and M) and enriched gene sets are highlighted in red.

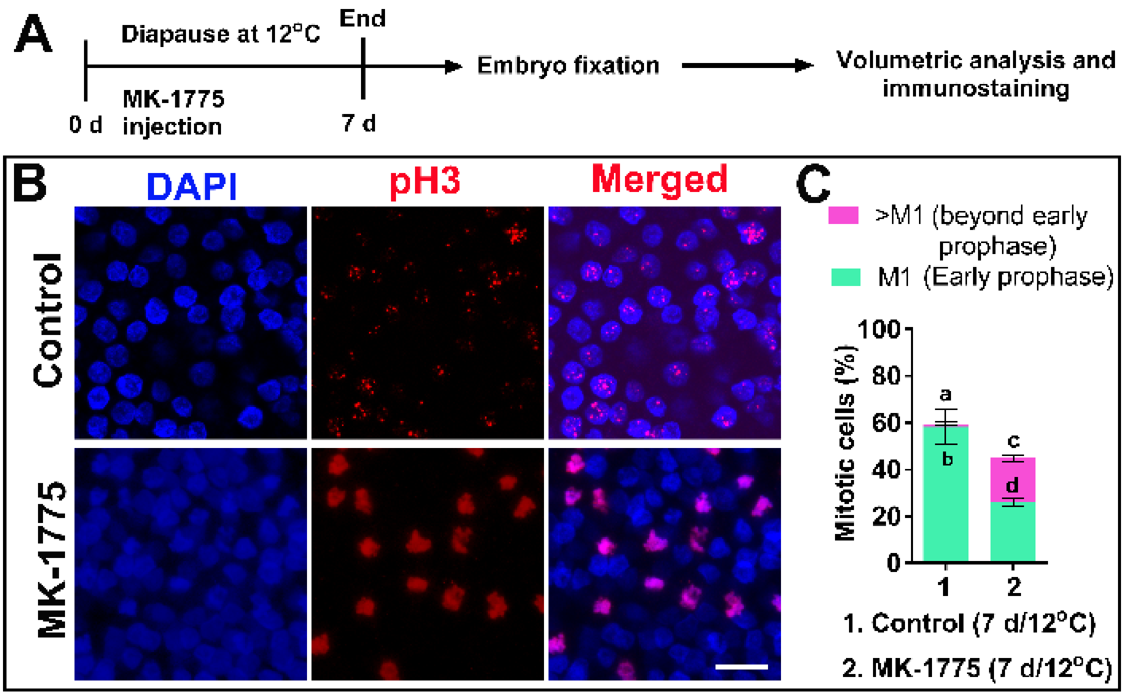

Figure S4. Investigation of the role of Wee1 in regulating the cell cycling of embryos in diapause at 12oC by treating embryos with the Wee1 kinase inhibitor MK-1775. (**A**) Experimental design. Fresh embryos were treated with MK-1775 and subsequently, they underwent diapause for 7 d at 12oC. PBS treated embryos under same condition were used as control. Following treatment, embryos were isolated, fixed, immunostained with anti-pH3 antibody and accessed for confocal microscopy. In a separate experiment, the treated and control embryos underwent volumetric analysis using 3-D image analysis method (Figure 8). (**B**) pH3 immunostaining of embryos shows majority of cells in early prophase in control embryos, whereas, following MK-1775 treatment, cell cycling progresses. Bars 20 µm. (**C**) Quantification of pH3 positive cells following MK-1775 show significantly higher number of cycling beyond early prophase. Different connecting letter refers that the compared groups are significantly different to each other (Two-way ANOVA; a vs. b: $p<0.0001$; c vs. d: $p=0.0207$; a vs. c: $p<0.0001$).

Table S1. Primer lists.

SN	Genes	Forward Primer	Reverse Primer
1.	GAPDH	ACCTGCATCTGCCCATTTGA	ACTGTCAAGGCTGAGAACGG
2.	Cyclin E1	TGGAGCCTGTACAGAAGATGAA	GGATGTGTCCACTAGCATGG
3.	Cyclin A1	TACAGCTGCCAAGAGGATGA	GGATGTGTCCACTAGCATGG
4.	Cyclin A2	GCTGGTGGAAGTTGGAGAAG	CCTCAGAACTTGCTTCTTGTTG
5.	Cyclin β2	GGACCTGGCAAGATGACAA	TTGTGGAGCAACACATCAGAG
6.	Wee1	AAGCATTCAGTGCTGCTGTC	CTTGCCAATGAGTCGAGATG
